# Subclinical systolic dysfunction detected by 2D speckle tracking echocardiography in adults with diabetes mellitus: systematic review and meta-analysis of 6668 individuals with diabetes mellitus and 7218 controls

**DOI:** 10.1007/s10554-023-02810-4

**Published:** 2023-03-30

**Authors:** Seyed-Mohammad Ghoreyshi-Hefzabad, Prajith Jeyaprakash, Ha Q. Vo, Alpa Gupta, Koya Ozawa, Faraz Pathan, Kazuaki Negishi

**Affiliations:** 1 Sydney Medical School Nepean, Faculty of Medicine and Health, Charles Perkins Centre Nepean, The University of Sydney, Kingswood, Australia; 2 Department of Cardiology, Nepean Hospital, Kingswood, NSW Australia; 3 Menzies Institute for Medical Research, University of Tasmania, Hobart, TAS Australia; 4 Present Address: The University of Sydney, Kingswood, NSW 2747 Australia

**Keywords:** Myocardial strain, 2D-speckle tracking echocardiography, Diabetes mellitus, Diabetic cardiomyopathy, Meta-analysis, Mean difference

## Abstract

**Purpose:**

Speckle tracking echocardiography (STE) can help to identify subclinical features of diabetic cardiomyopathy (DCM). There is, however, significant heterogeneity in the reported strain values in literature. We performed a systematic review and meta-analysis to compare cardiac systolic strain values assessed by 2D-STE in asymptomatic adults with diabetes mellitus (DM) and healthy controls.

**Methods:**

Five databases were searched, and a total of 41 valid studies (6668 individuals with DM and 7218 controls) were included for analysis. Pooled mean in each group and mean difference (MD) for left ventricular global longitudinal strain (LVGLS), LV global circumferential strain (LVGCS), LV global radial strain (LVGRS), LV longitudinal systolic strain rate (LVSR), left atrial reservoir strain (LARS) and right ventricular GLS (RVGLS) were assessed.

**Results:**

Patients with DM had overall 2 units lower LVGLS than healthy subjects 17.5% [16.8, 18.3], vs 19.5 [18.7, 20.4], MD = − 1.96 [− 2.27, − 1.64]. Other strain values were also lower in patients with DM: LVGCS (MD = − 0.89 [− 1.26, − 0.51]); LVGRS (MD = − 5.03 [− 7.18, − 2.87]); LVSR (MD = − 0.06 [− 0.10, − 0.03]); LARS (MD = − 8.41 [− 11.5, − 5.33]); and RVGLS (MD = − 2.41 [− 3.60, − 1.22]). Meta-regression identified higher body mass index (BMI) as the single contributor to worse LVGLS, LVGCS and LVSR. Those with higher Hemoglobulin A1c had worse RVGLS.

**Conclusion:**

Myocardial strains were reduced in whole heart in patients with DM. The largest reduction was observed in LA reservoir strain, followed by RVGLS and LVGLS. Higher BMI in patients with DM is associated with worse LV strain values.

**Supplementary Information:**

The online version contains supplementary material available at 10.1007/s10554-023-02810-4.

## Introduction

Diabetes mellitus (DM) is one of the most prevalent chronic diseases in the world [1], and contributes to significant cardiac mortality and morbidity [2, 3]. The risk of heart failure increases at least 2–5 times in patients with DM [2, 3]. Diabetic cardiomyopathy (DCM) is defined as the development of myocardial dysfunction in individuals with DM, independent of coronary artery disease, hypertension, valvular, or congenital heart disease [3]. Although most of the previous studies using conventional echocardiography have emphasized left ventricular (LV) diastolic dysfunction as the earliest and main functional alteration in the course of DCM [4, 5, 6, 7], some recent studies using speckle tracking echocardiography (STE) have reported subclinical systolic dysfunction in adults with DM predates the development of LV diastolic dysfunction [8]. Early detection of subclinical and reversible cardiac dysfunction in patients with DM using STE would lead to treatment, which could prevents subsequent development of heart failure[9].

Investigators have tried to clarify the impact of DM on cardiac mechanics using STE recently [10, 11, 12]. Most of these studies reported impaired global longitudinal strain (GLS) in asymptomatic patients with DM [13, 14, 15, 16]. However, the current data are still conflicting and non-homogeneous. Some studies reported similar GLS between individuals with DM and controls [10, 17, 18, 19]. The measured GLS in patients with DM in some studies are higher than measured GLS in healthy controls of some other studies [20, 21, 22]. In addition, the exact extent of the decrease and alteration of left ventricle GLS (LVGLS) and alteration of strain in other directions (circumferential and radial) in individuals with DM have been less studied [10, 20, 23]. Finally, assessment of left atrial (LA) and right ventricle (RV) mechanics using STE in asymptomatic patients with DM is a new era of interest [12, 24].

Thus, we aimed (1) to conduct a systematic review on the strain values of LV, LA and RV assessing by 2D-STE in asymptomatic adults with DM and healthy controls; (2) to synthesize the information qualitatively; (3) to perform quantitative analysis using meta-analysis to estimate the pooled mean difference (MD) of these strain values in individuals with DM and controls; and (4) to clarify possible sources of variation affecting the strain values by meta-regression analysis.

## Methods

### Search strategy

We performed a systematic review and meta-analysis following the PRISMA (Preferred Reporting Items for Systematic review and Meta-Analysis) guideline. Under the guidance of a librarian at the University of Sydney, we searched five databases (MEDLINE, Embase, Scopus, Web of Science and Cochrane central register of controlled trials) for the key terms of “myocardial strain/ LV, RV, LA/function, dysfunction”, “speckle tracking echocardiography, deformation imaging/analysis” and “diabetes mellitus”. The search was limited to human articles published in English and completed on March 30, 2020. Search hedges created are listed in the Online Supplementary Materials (Appendix A). The reference lists of relevant studies were manually searched for any possible additional appropriate study. The study was prospectively registered with the PROSPERO database of systematic reviews (Subclinical systolic dysfunction detected by 2D speckle tracking echocardiography in diabetes mellitus: a systematic review and meta-analysis; CRD42020197825).

### Study selection

From these lists, studies were included if the articles reported strain values using 2D-STE in asymptomatic patients with DM and control group. Two independent investigators (S.G and A.G) reviewed and chose studies if the articles met the following criteria: (1) studies reported strain values of LV and/or LA and/or RV in adult patients with DM (type 1 or 2), (2) studies included a control group, 3) were > 18 years of mean age. The definition of each group and exclusion criteria varies with the studies and are shown in Online Supplementary Materials (Online Table S1). If one study had multiple groups of patients or controls, we selected the lower risk group for our meta-analysis to avoid extreme cases. When multiple studies that used the same data set were identified, the largest study was included for assessment and analysis.

### Study exclusion

Our exclusion criteria were reduced ejection fraction, presence of known coronary artery disease (CAD), or any structural heart disease. Multiple studies used different methods to exclude CAD patients (e.g. presence of known history or symptoms of CAD, positive non-invasive investigations). Detailed exclusion criteria of each study are shown in Supplementary Table S1. We also excluded studies in which strain was calculated using 3D-STE, Doppler tissue imaging, or cardiac magnetic resonance imaging. In addition, case reports, conference presentations, review articles, editorial, and expert opinions were excluded.

### Data collection

All demographic, ultrasound system and software, common clinical characteristics, and strain information were extracted from texts, tables, and graphs and summarized into a standardized extraction sheet. Authors of eligible studies were contacted by e-mail to obtain missing information.

### The outcome of interest

In this meta-analysis, our outcomes of interest were LV, LA and RV strains (LVGLS, LV global circumferential strain (LVGCS), LV global radical strain (LVGRS), LV longitudinal systolic strain rate (LVSR), LA reservoir strain, and RVGLS) measured by 2D-STE in adults with DM and control groups.

### Statistical analysis

The pooled means and 95% confidence interval (CI) of LVGLS, LVGCS, LVGRS, LVSR, LA reservoir strain, and RVGLS in patients with DM and control groups were computed using random-effects models weighted by inverse variance and are showed in the forest plot. Although our primary outcome was MD, we also calculated standardized mean difference (SMD, also known as Cohen’s D) in each study and pooled to compare the effect sizes among various strains with different normal ranges[25, 26]. SMD = 0.2 is considered as a small effect size, SMD = 0.5 as a ‘medium’ effect size, and SMD = 0.8 as a large effect size [25]. The heterogeneity between studies were assessed by the Cochran Q test and the inconsistency factor (I^2^). I^2^ values of 25%, 50%, and 75% corresponded to a low, moderate, and high degree of heterogeneity, respectively. Potential publication bias was assessed using Funnel plots with and without the Duval and Tweedie trim and fill methodology, and the Egger’s test. Meta-regression analysis was performed for variables that were reported in > 50% of studies to assess possible study factors associated with heterogeneity. The beta coefficient and its CIs were derived using the least-mean squares fitting method. Sensitivity analyses were performed to examine the effect of methodological diversity (definition of study groups based on the inclusion of hypertensive patients) on the overall pooled estimates. Statistical analysis was performed using R version 4.0.0 and RStudio version 1.2.5042 (The R Foundation for Statistical Computing, Vienna, Austria) with the “metafor” package. Two-tailed p values were used, and the threshold of statistical significance was 0.05 except for the Egger test, where 0.1 was applied. Based on the EACVI/ASE/Industry taskforce recommendation [27] and to avoid unnecessary confusion, we considered the absolute value of each strain value.

## Results

### Study selection

**Fig. 1:**
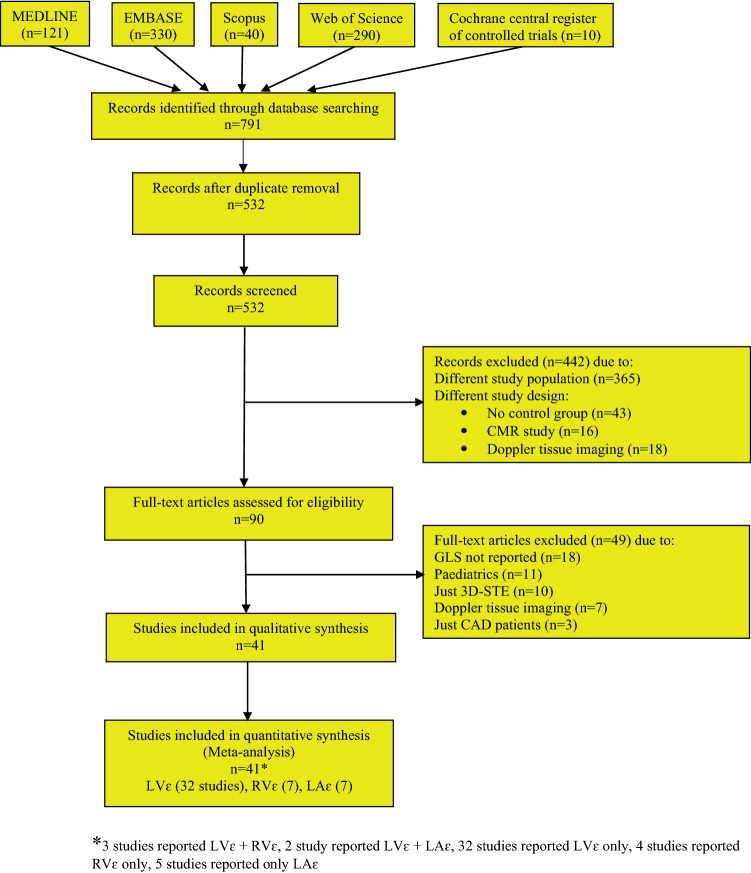
PRISMA Flow Chart this flow chart illustrates the selection process for published reports on LV, LA and RV strains (LVGLS, LVGCS, LVGRS, LVSR, LA reservoir strain and RVGLS) measured by 2D-STE in adult with DM and control groups. *GLS* global longitudinal strain; *GCS* global circumferential strain; *GRS* global radial strain; *LVSR* left ventricular longitudinal systolic strain rate; *LV* left ventricular; *RV* right ventricular; *DM* diabetes mellitus

Figure 1 shows PRISMA flowchart of our study. Our search strategy revealed 791 results from 5 databases (MEDLINE [n = 121], EMBASE [n = 330], Scopus [n = 40], Web of Science [n = 290], Cochrane central register of controlled trials [n = 10]). Following the removal of 259 duplicates, the titles and abstracts of 532 articles were screened for eligibility. Four hundred and forty-two studies were excluded because of the different study populations and different study designs (no control group, CMR study, Doppler tissue imaging). Ninety full-text articles assessed for eligibility. An additional 49 studies were excluded for the following reasons: no GLS data, Doppler tissue imaging, just 3D-STE results, paediatrics, and patients with coronary artery disease. Finally, 41 valid studies (6668 individuals with DM and 7218 controls) met the selection criteria and were included in this meta-analysis. Thirty-two studies were eligible for LVGLS, 14 for LVGCS, 9 for LVGRS, 13 for LVSR, 7 for LA reservoir strain, and 7 for RVGLS. Articles included were published from 2009 to 2020. Most of the studies used age and gender-matched healthy subjects for the control group. Summary of included studies is shown in Table 1. Further detailed information can be found in online Supplementary Materials (Tables S2 and S3).

**Table 1:** Summary of included studies

First Author	Year	DM (n)	Control (n)	Software	DM Type	Age Mean ± SD (DM)	Age Mean ± SD (Control)	Female % (DM)	Female % (Control)	BMI Mean ± SD (DM)	BMI Mean ± SD (Control)	HTN % (DM)	HTN % (Control)	strain	Chamber
Nakai [40]	2009	60	25	EchoPAC	2	63 ± 12	62 ± 11	43	40	NA	NA	78	0	L/C/R	LV
NG [41]	2009	47	53	EchoPAC	2	58 ± 5.5	56.2 ± 6.6	0	0	28 ± 3.2	26 ± 3	NA	NA	L/C/R/SR	LV
Ernande [8]	2011	114	88	EchoPAC	2	52 ± 4.5	51.7 ± 2.6	39.4	65.9	29 ± 5	24 ± 3	NA	NA	L/R	LV
Mondillo [42]	2011	34	36	EchoPAC	2	64 ± 12.3	62.3 ± 12.7	41.2	52.8	27.4 ± 4.2	23.8 ± 2.4	0	0	Reservoir	LA
D'Andrea [43]	2012	45	35	EchoPAC (AFI)	2	56.3 ± 8.2	55 ± 9.3	40	48.5	28.3 ± 3.2	26.6 ± 4.5	0	0	L	LV
Kadappu [44]	2012	73	73	EchoPAC	2	43 ± 11	43 ± 10	NA	NA	NA	NA	59	0	Reservoir	LA
Conte [45]	2013	44*	24	EchoPAC	2	60.9 ± 6.6	58.4 ± 9.4	47	45	25.7 ± 1.9	23.5 ± 1.5	19	0	L/C/R/SR	LV
Tadic [46]	2014	60	60	EchoPAC	2	54 ± 7	51 ± 8	50	51	28.1 ± 2.7	24.4 ± 2.6	0	0	Reservoir	LA
Zoroufian [47]	2014	39	37	EKO 7	2	55.7 ± 8.48	51.5 ± 8.1	69.2	54.1	29 ± 4.7	27 ± 3.3	0	0	L/SR	LV
Bakirci [48]	2015	132	80	EchoPAC	2	54.5 ± 9.6	53.2 ± 9	42.4	37.5	23.5 ± 5.8	22.1 ± 6.1	0	0	Reservoir	LA
Enomoto [28]	2015	74	24	2D wall motion tracking	2	51 ± 15	49 ± 12	56.7	33.3	23.8 ± 5.2	23.1 ± 2.9	0	0	L	LV
Jensen [11]	2015	1065	198	EchoPAC	1	49.5 ± 14.5	48.7 ± 14	47.9	48	25.5 ± 3.9	24.5 ± 3.2	NA	NA	L	LV
Karagov [49]	2015	82	90	EchoPAC	2	54.3 ± 10	55.1 ± 2.4	56.1	52	29 ± 4.7	29 ± 9.3	NA	NA	L	LV
Skali [50]	2015	1322	1742	TomTec	2	NA	NA	60.7	60.8	NA	NA	91.2	71	L	LV
Tadic [12]	2015	57	54	EchoPAC	2	54 ± 7	51 ± 8	49	54	28 ± 2.8	24 ± 2.5	0	0	L	LV,RV
Tadic [51]	2015	50	50	EchoPAC	2	52 ± 8	50 ± 7	48	52	27 ± 2.5	24 ± 2.2	0	0	L/C/R/SR	LV
Abdel-Salem [52]	2016	30	30	EchoPAC	1	26.5 ± 4.3	28.8 ± 4.7	63.3	53.3	27.9 ± 7.2	25.7 ± 3.1	0	0	L/SR	LV
Bakhum[53]	2016	60	30	MyLab 60 Xvision	1	21.08 ± 5.7	23.6 ± 6.62	53.3	43.3	22.87 ± 3.8	25.68 ± 3.83	0	0	L/C/SR	LV
Jedrzejewska [24]	2016	50	50	EchoPAC	1	30.7 ± 7.2	27.3 ± 4.9	50	48	24 ± 3.5	22 ± 2.6	0	0	L/C/R	LV,RV
Jorgensen [21]	2016	770	234	EchoPAC	2	NA	NA	39.6	35.5	NA	NA	NA	NA	L	LV
Loncarevic [23]	2016	70	80	X-strain	2	54.8 ± 7.7	54.8 ± 4.9	45.7	45	27.5 ± 3.4	25.7 ± 3.7	0	0	L/C/SR	LV
Mochizuki [54]	2016	137†	69	EchoPAC	2	55 ± 15	52 ± 16	56	62	24 ± 4	21 ± 3	40	0	L/Reservoir	LV, LA
Tadic [55]	2016	42	40	EchoPAC	2	54 ± 8	51 ± 8	48	50	27.1 ± 2.3	26.1 ± 2.1	0	0	L	RV
Kishi [22]	2017	368‡	1485	2D wall motion tracking	2	50.6 ± 3.7	49.8 ± 3.7	53.8	64.7	35.8 ± 8.4	28.5 ± 6.6	72.6	23.7	L/C	LV
Suto [16]	2017	145	90	EchoPAC	2	60 ± 13	57 ± 15	46	56	25 ± 5	22 ± 3.9	58.6	0	L	LV
Tadic [56]	2017	55	50	EchoPAC	2	54 ± 7	48 ± 8	53	54	28.4 ± 3	24.5 ± 2.4	0	0	Reservoir	LA
Tadic [57]	2017	59	45	EchoPAC	2	54 ± 1	49 ± 1.3	47	47	29.3 ± 0.4	24.3 ± 0.4	0	0	L	RV
Vukomanovic [58]	2017	50	40	EchoPAC	2	55 ± 7	50 ± 9	48	45	29 ± 3.3	24 ± 2.8	0	0	L/C/R	LV
Ahmed [59]	2018	39	15	EchoPAC	1	18.2 ± 1.7	18.8 ± 2.3	66.6	66.6	26.2 ± 3.9	22.8 ± 3.3	0	0	L	RV
Jorgensen [10]	2018	57§	80	EchoPAC	2	NA	NA	43.9	35	NA	NA	0	0	L/C/SR	LV
Lin [20]	2018	505	1416	EchoPAC	2	57.1 ± 10	46.5 ± 10.2	33.9	41.5	25.8 ± 4	23.4 ± 3.3	44.4	10	L/C	LV
NG [37]	2018	337	316	EchoPAC	2	57 ± 12	57 ± 14	36.8	37.4	29 ± 5.6	28 ± 4.9	56.4	27.5	L/SR	LV
Philouze [19]	2018	44	35	EchoPAC	2	56 ± 6	52 ± 7	41	51.4	26.9 ± 3.2	24.2 ± 3.6	41	17	L/SR	LV
Ringle [60]	2018	66	26	TomTec	1	37.6 ± 9	35.1 ± 7	71	69	24 ± 3	23 ± 3	0	0	L/C/R/SR	LV
Stevanovic [13]	2018	121	41	EchoPAC	2	54.9 ± 7.3	52.6 ± 5.2	19	29	28 ± 5	27 ± 3.8	0	0	L	LV
Tadic [61]	2018	48	44	EchoPAC	2	55 ± 9	52 ± 8	47.9	50	27 ± 2.3	26 ± 2.2	0	0	L/C/R	LV
Berceanu [62]	2019	60	90	EchoPAC	1	25 ± 6	30 ± 8	33.3	34.4	27 ± 6	23 ± 4	NA	NA	L	RV
Bogdanovic [14]	2019	20||	20	EchoPAC	2	43.2 ± 3.6	36.2 ± 2	50	60	24.6 ± 1	23.8 ± 0.7	0	0	L	LV
Cameli [17]	2019	52	60	EchoPAC	2	59.3 ± 14.9	59.8 ± 9.1	42.3	65	27 ± 5.3	25 ± 4.9	0	0	L/Reservoir	LV, LA
Haley [15]	2020	151	146	EchoPAC	2	23.5 ± 1.4	22.5 ± 4.1	63.6	56.8	36.6 ± 9	23.3 ± 3.2	NA	NA	L/C/SR	LV
Roberts [18]	2020	34	17	EchoPAC	1	42 ± 13	41 ± 13	29	35	27 ± 4	25 ± 3	29	6	L/SR	LV,RV

[*, †, ‡, §, ||] are standard operator symbols for search criteria for meta analysis

* stands for "wild card search"

### LV strain in adults with DM vs controls

**Fig. 2:**
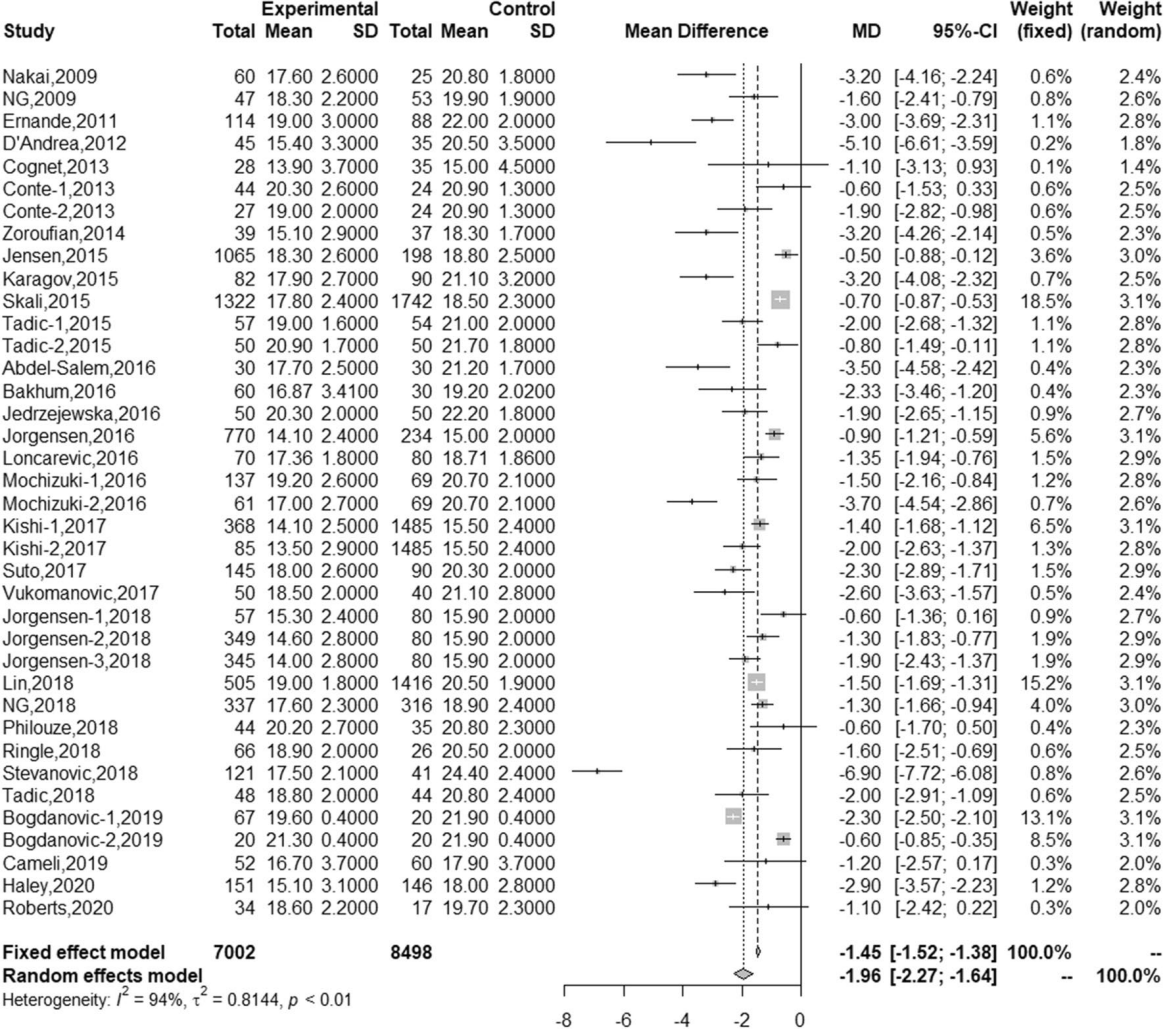
Forest plot for LVGLS. *GLS* global longitudinal strain; *GCS* global circumferential strain; *GRS* global radial strain; *LVSR* left ventricular longitudinal systolic strain rate; *LV* left ventricular; *RV* right ventricular; *DM* diabetes mellitus

All LV strain values (GLS, GCS, GRS, and LVSR) were reduced in adults with DM compared to healthy subjects. Patients with DM had significantly lower LVGLS than healthy subjects (17.5% [16.8, 18.3] vs 19.5% [18.7, 20.4]) with MD of − 1.96% [− 2.27, − 1.64]) (Fig. 2 and Table 2). LVGCS, LVGRS, and LVSR were also lower in individuals with DM, but the effect sizes were small (Table 2, Online Figures S1–S3). Although no significant publication bias was identified by the funnel plot with and without Trim and Fill (Online Figures S4–S7) and the Egger’s test (except for LVGCS of controls and LVGRS of patients with DM), there were a high degree of heterogeneity in all LV strain values. Therefore, a univariate meta-regression was performed to find factors that have significant contributions to the heterogeneity (Table 3). It revealed that increasing body mass index (BMI) was associated with worse LVGLS, LVGCS, and LVSR. In addition, studies that used Wall Motion Tracking software [22, 28] had reported significantly lower LVGLS and LVGCS compared to EchoPAC software (β for LVGLS of DM = − 3.79 [− 7.05, − 0.53], *p* = 0.02; β for LVGCS of DM = − 5.17 [− 10.14, -0.2], *p* = 0.04).

**Table 2:** Main results of meta-analysis

Strain variable	Studies (n)	DM (n)	Control (n)	Mean [95% CI] in DM	Mean [95% CI] in Control	MD [95% CI] Random Effects model	Standardized MD [95% CI]
LVGLS	32	6114	6729	17.9 [17.1, 18.4]	19.8 [19.1, 20.5]	− 1.98 [− 2.46, − 1.51]	− 0.8 [− 1.0, − 0.7]
LVGCS	14	1626	3549	20.3 [18.6, 21.9]	21.3 [19.6, 22.9]	− 0.96 [− 1.48, − 0.45]	− 0.3 [-0.5, − 0.1]
LVGRS	9	529	400	42.7 [39.7, 45.6]	47.0 [43.1, 50.9]	− 4.0 [− 5.50, − 2.52]	− 0.4 [− 0.5, − 0.2]
LVSR	13	1029	924	1.0 [0.9, 1.1]	1.1 [1.0, 1.2]	− 0.07[− 0.13, − 0.02]	− 0.4 [− 0.7, − 0.1]
LA reservoir strain	7	543	428	28.0 [24.4, 31.6]	36.5 [34.0, 39.0]	− 8.42[− 11.6, 5.25]	− 1.2 [− 1.5, − 0.9]
RVGLS	7	341	311	23.8 [20.1, 27.4]	26.0 [23.4, 28.6]	− 2.38 [− 4.67, − 0.09]	− 1.1 [− 2.3, 0.1]

**Table 3:** Meta-regression results

Variable		DM type (II vs I)	Age, per 1 year	% Female, per 1%	SBP, per 1 mmHg	DBP, per 1 mmHg	HR, per 1 pm	%HTN, per 1%	BMI, per 1 kg/m2	HbA1C, per 1%
LV GLS	N	32	32	32	28	26	20	26	31	25
	β [95% CI]	− 0.8 [− 2.9, 1.2]	− 0.01 [− 0.1, 0.1]	− 0.02 [− 0.1, 0]	0.08 [− 0, 0.2]	0.05 [− 0.2, 0.3]	0 [− 0.2, 0.2]	0 [− 0, 0]	− **0.31 [**− **0.5, **− **0.1]**	− 0.56 [− 1.4, 0.3]
	*P-*value	0.44	0.45	0.54	0.16	0.65	0.97	0.72	**0.003**	0.19
LV GCS	N	14	14	14	12	11	8	12	13	12
	β [95% CI]	− 3.68 [− 7.9, 0.5]	0.02 [− 0.1, 0.2]	− 0.04 [− 0.2, 0.1]	0.06 [− 0.2, 0.4]	− 0.25 [− 1, 0.5]	− 0.2 [− 1.3, 0.9]	− 0.03 [− 0.1, 0]	− **0.67 [**− **0.9, -0.5]**	− 1.4 [− 4, 1.2]
	*P-*value	0.08	0.8	0.48	0.71	0.51	0.72	0.34	** < 0.0001**	0.3
LV GRS	N	9	9	9	8	7	6	7	8	7
	β [95% CI]	1.44 [− 6.3, 9.2]	0.01 [− 0.3, 0.3]	− 0.04[− 0.2, 0.1]	0 [− 0.5, 0.5]	− 0.35 [− 1.5, 0.8]	0.69 [− 0.5, 1.9]	0.05 [− 0.1, 0.2]	0.21 [− 1.7, 2.1]	3.8 [− 0.8, 8.4]
	*P-*value	0.71	0.97	0.62	0.99	0.54	0.25	0.33	0.83	0.1
LV LSSR	N	13	13	13	11	10	7	11	13	11
	β [95% CI]	− 0.13 [− 0.3, 0]	0 [0, 0]	0 [0, 0]	0 [0, 0]	0 [0, 0]	0 [0, 0]	0 [0, 0]	− **0.02 [**− **0.1, 0]**	− 0.01 [− 0.1, 0.1]
	*P-*value	0.06	0.67	0.97	0.81	0.82	0.9	0.88	**0.01**	0.89
RV GLS	N	7	7	7	7	7	4	6	7	7
	β [95% CI]	0.32 [− 4.8, 5.4]	0.13 [− 0.1, 0.3]	− **0.23 [**− **0.4, 0]**	0.19 [− 0.4, 0.8]	− **0.83 [**− **1.2, **− **0.5]**	− 0.34 [− 1.2, 0.5]	0.15 [− 0.1, 0.4]	− 0.74 [− 2.2, 0.7]	− **5.88 [**− **10.1, -1.7]**
	*P-*value	0.9	0.15	**0.02**	0.5	** < 0.001**	0.45	0.21	0.33	**0.006**
LA reservoir strain	N		7	6	6	5	5	7	6	3
	β [95% CI]		0.07 [− 0.7, 0.8]	0.52 [− 0.1, 1.2]	0.2[− 0.7, 1.1]	0.13 [− 1.5, 1.4]	0.38 [− 1.9, 2.7]	− 0.07 [− 0.3, 0.1]	1.05 [− 0.7, 2.8]	− 1.1 [− 12, 9.8]
	*P-*value		0.84	0.1	0.65	0.84	0.74	0.46	0.23	0.84

Bold is statistically significant p-value (p<0.05)

### LA strain in adults with DM vs controls

**Fig. 3:**
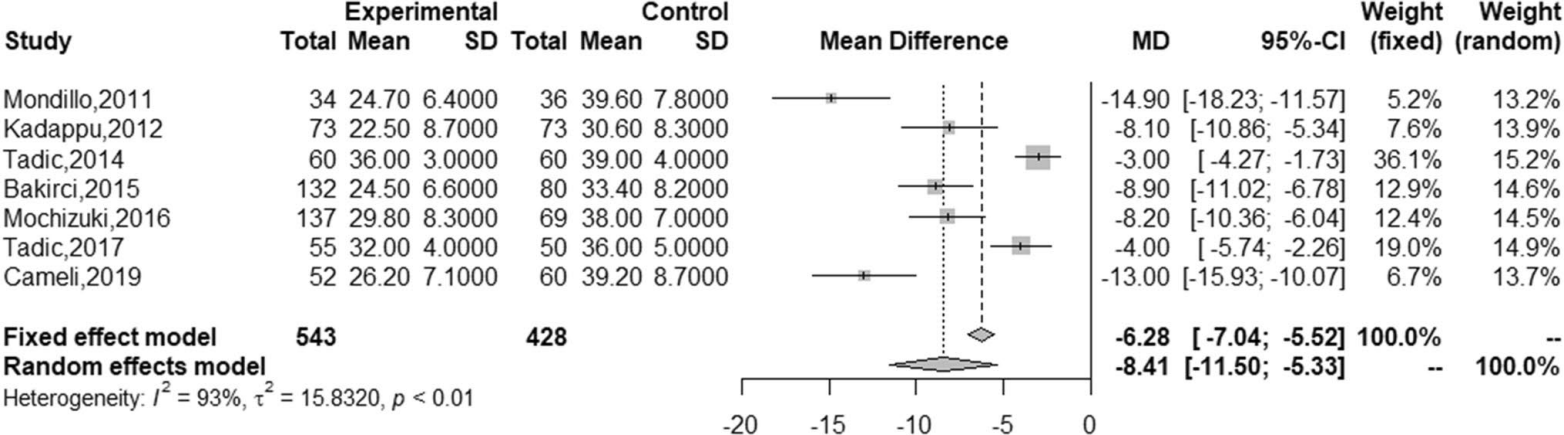
Forest plot for LA reservoir strain. *GLS* global longitudinal strain; *GCS* global circumferential strain; *GRS* global radial strain; *LVSR* left ventricular longitudinal systolic strain rate; *LV* left ventricular; *RV* right ventricular; *DM* diabetes mellitus

LA reservoir strain was significantly lower in individuals with DM compared to healthy controls (28.0% [23.9, 32.1] vs 36.5 [34.2, 38.9]) with a large MD of − 8.41 [− 11.5, − 5.33]) (Fig. 3 and Table 2). Egger’s test showed a significant publication bias in patients with DM (p = 0.02). Although a high degree of heterogeneity was identified in adults with DM (I^2^ = 98.59%), meta-regression could not find any significant contributor to this heterogeneity (Table 3). Funnel plots for LA reservoir strain with and without Trim and Fill in adults with DM and control groups are shown in the online Fig. S8.

### RV strain in adults with DM vs controls

**Fig. 4:**
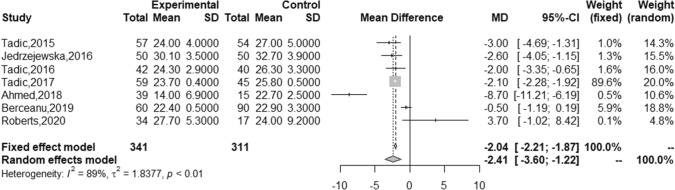
Forest plot for RVGLS. *GLS* global longitudinal strain; *GCS* global circumferential strain; *GRS* global radial strain; *LVSR* left ventricular longitudinal systolic strain rate; *LV* left ventricular; *RV* right ventricular; *DM* diabetes mellitus

RVGLS was also significantly lower in adults with DM (24.1% [23.0, 25.1] vs 26.0 [24.0, 28.0]) with an MD of − 2.41 [− 3.60, − 1.22]) (Fig. 4 and Table 2*).* Although there was no significant publication bias in the DM group, a high degree of heterogeneity was identified (I^2^ = 98.89%). We found that female proportion, diastolic blood pressure, and haemoglobin A1c (HbA1C) were the factors linked to this heterogeneity in patients with DM (Table 3). Funnel plots for RVGLS with and without Trim and Fill in individuals with DM and control groups are shown in Fig. S9.

### Additional analysis

Sensitivity analyses based on the inclusion or exclusion of hypertensive patients in each study revealed no obvious effects of hypertension on LVGLS (Online Figure S10).

In the present study, in order to evaluate DCM patients, we initially excluded studies that reported strain values in patients with DM and CAD in the main analyses. There were 4 studies [23, 29, 30, 31] that reported LVGLS in patients with DM and CAD. Table 4 summarizes these studies. The pooled mean LVGLS in patients with both DM and CAD was 16.4% [15.2, 17.6] (Online Figure S11), whereas the pooled mean of LVGLS in patients with DM without CAD was 17.8 [17, 18.6].

**Table 4:** Summary of studies that reported mean LV GLS ± SD in patients with DM and CAD

First Author	Year	DM (−) CAD (−)	DM (−) CAD ( +)	DM ( +) CAD (−)	DM ( +) CAD ( +)
Zuo [29]	2015	–	17.32 ± 2.27 (n = 40)	–	16.65 ± 2.29 (n = 33)
Loncarevic [23]	2016	18.71 ± 1.86 (n = 80)	–	17.36 ± 1.80 (n = 70)	16.26 ± 2.84 (n = 70)
Rasalingam [30]	2016	–	–	18.5 ± 4.0 (n = 45)	18.0 ± 2.5 (n = 39)
Wierzbowska-Drabik [31]	2018	–	17.4 ± 4.0 (n = 85)	–	14.5 ± 3.6 (n = 42)

## Discussion

This is the first systematic review and meta-analysis of the pooled difference of cardiac strain values assessed by 2D-STE in 6,668 asymptomatic patients with DM compared to 7218 healthy controls from 41 studies. There were three major findings. First, systolic strain values are significantly reduced in asymptomatic patients with DM, not only in the LV but also in the LA and RV. This confirms that DCM is a global cardiac phenomenon and not merely an LV dysfunction. Second, LA reservoir strain, RVGLS, and LVGLS had large effect sizes with SMD > 0.8. Finally, higher BMI associated with worse LVGLS, LVGRS, and LVSR, whereas higher HbA1c is the main contributor to worse RVGLS in patients with DM.

Our meta-analysis confirmed that subclinical cardiac dysfunction in DCM is not limited to the LV but also seen in the RV and LA. The effect size assessed by SMD was the largest in LA reservoir strain (SMD − 1.2 [− 1.5, − 0.9]), closely followed by RVGLS (− 1.1 [− 1.9, − 0.2]) and LVGLS (− 0.8 [− 1.0, − 0.7]). Larger effect sizes in the LA and RV further support the whole heart dysfunction in DCM. The clear separation of LA reservoir strain between patients with DM (upper limit of 95%CI of 32.1%) and control (lower limit of 95%CI of 34.2%) (Table 2) indicates that LA reservoir strain can be a good diagnostic parameter. Similar separation was observed in LVGLS, where lower limit of LVGLS in control was 18.7% and upper limit of DM was 18.3%. On contrary, the reduction in LVGCS was small by − 0.89% [− 1.26, − 0.51] although it was still statistically significant. The reason for the discrepancy in effect sizes between LVGLS and LVGCS is still unclear [32, 33, 34]. Some studies argued that in early stages of myocardial dysfunction, impairment in longitudinal deformation accompanies by a compensatory increase in circumferential deformation to preserve of gross LVEF [34]. The decline in circumferential deformation only occurs in advanced stages and ultimately results in falling LVEF [35]. However, our meta-analysis showed that asymptomatic patients with DM with normal LVEF have impaired LVGCS and LVGRS in addition to the impaired LVGLS. This corroborates our previous findings in 3D STE, where DCM is at least pan-LV phenomena rather than reduction in a single direction [36]. More studies are warranted to reveal the changes in multiple directions of myocardial strains during the evolvement of DCM.

Our meta-regression showed that BMI is the only significant source of heterogeneity in subclinical LV systolic dysfunction measured by 2D-STE in patients with DM. We confirmed that increasing BMI was associated with worse LVGLS, LVGCS, and LVSR in patients with DM. The impact of obesity on LV function in adults with DM has been investigated in some studies and they were also included in the present meta-analysis [15, 16, 37]. These studies showed that increasing BMI and DM are independent predictors of impaired LV myocardial systolic dysfunction. Ng et al. reported that not only the combination of DM and higher BMI has an additive detrimental effect on LV myocardial function, but also increasing BMI per se is a stronger determinant of impaired LV myocardial function than DM [37]. On the other hand, we found that increasing HbA1c, as the main source of heterogeneity in RVGLS, was significantly associated with worse RVGLS in both adults with DM and control groups.

Little is known that the additive effects of concomitant CAD on myocardial deformation of DCM. Four studies reported strain values in patients with DM and CAD [23, 29, 30, 31] (Table 4). Although from limited number of articles, LV GLS of less than 17 may suggest the possibility of concomitant CAD.

Based on convincing results of EMPA-REG OUTCOME [38] study and other similar trials [39], 2019 ESC Guidelines on DM, pre-DM and cardiovascular diseases (CVD) recommended the use of sodium-glucose co-transporter 2 (SGLT2) inhibitors in patients with T2DM and CVD or at high/very high cardiovascular (CV) risk to reduce CV events. Recent data suggest that these relatively new glucose-lowering drugs can prevent heart failure in patients with DM. However, potential candidates of SGLT2 inhibitors are not clearly defined. Our systematic review and meta-analysis showed that 2D-STE can be helpful in the diagnosis of subclinical DCM in early stages. Therefore, patients with lower strain values in 2D-STE can be potential candidates to treat with SGLT2 inhibitors to prevent and treat subsequent clinical heart failure.

### Study limitations

Several factors merit consideration in the interpretation of our results. First, like all meta-analyses, this study is limited by quality in the original studies and publication bias, although we used standard approaches to detect this. In addition, observational studies may be limited by biases in the recruitment process. Second, we have assumed that all the measurements were performed by the experts, but the levels of expertise among individuals who have measured the strain are uncertain. Third, significant heterogeneities among studies were identified. Thus, we performed subsequent meta-regression analyses to explain the sources of the variations. Fourth, this study does not have information about right atrial strain values. Fifth, included studies did not outline information regarding duration of DM in the recruited patients, and therefore could not be assessed for impact on heterogeneity. Sixth, this study did not examine impaired left ventricular diastolic performance, which is thought to occur early in diabetic cardiomyopathy. Finally, our study may not have enough power to test vendor differences because only eight studies reported other than EchoPAC software data.

## Conclusion

Subclinical DCM can be detected by 2D-STE. Cardiac mechanics is impaired in all directions in patients with DM and exists in the LV, LA, and RV. The standardized reduction of strain was the largest in LA reservoir strain, closely followed by RVGLS and LVGLS. Higher BMI in adults with DM is associated with worse LV strain values, and higher HbA1c is associated with worse RVGLS.

## Supplementary Information

Below is the link to the electronic supplementary material.

Supplementary file1 (DOCX 9195 KB)

## Abbreviations

DCM: Diabetic cardiomyopathy
LVGLS: Left ventricular global longitudinal strain
LVGCS: Left ventricular global circumferential strain
LVGRS: Left ventricular global radial strain
LVSR: Left ventricular longitudinal systolic strain rate
MD: Mean difference
RVGLS: Right ventricular global longitudinal strain
STE: Speckle tracking echocardiography
DM: Diabetes mellitus
