# Supplementary material for: Subclinical systolic dysfunction detected by 2D speckle tracking echocardiography in adults with diabetes mellitus: systematic review and meta-analysis of 6668 individuals with diabetes mellitus and 7218 controls
Source: Int J Cardiovasc Imaging. 2023 Mar 30;39(5):977–89. doi: 10.1007/s10554-023-02810-4 (PMC10160195; doi:10.1007/s10554-023-02810-4)

**Online supplementary Materials**

**Appendix A – Search criteria for the meta-analysis**

**Appendix B – Supplementary Tables**

Table S1: Definition of study groups of included studies

Table S2: Ultrasound system information of included studies

Table S3: Summary of included articles

Table S4: Meta-regression results in control groups

Table S5: Summary table with GE vendor only

**Appendix C – Supplementary Figures**

Figure S1: Forest plots for LVGCS

Figure S2: Forest plots for LVGRS

Figure S3: Forest plots for LVSR

Figure S4: Funnel plots with Trim and Fill plots for LVGLS

Figure S5: Funnel plots with Trim and Fill plots for LVGCS

Figure S6: Funnel plots with Trim and Fill plots for LVGRS

Figure S7: Funnel plots with Trim and Fill plots for LVSR

Figure S8: Funnel plots with Trim and Fill plots for LA reservoir strain

Figure S9: Funnel plots with Trim and Fill plots for RVGLS

Figure S10: Forest plots of LVGLS after exclusion of studies with HTN patients

Figure S11: Forest plot of LVGLS in CAD patients who have diabetes mellitus

**Appendix A – Search criteria for the meta-analysis on 30/03/2020**

**Ovid MEDLINE(R) ALL (1946 to March 26, 2020)**

| **#** | **Search Statement** | **Results** |
| --- | --- | --- |
| 1 | myocardial strain.mp. | 1118 |
| 2 | deformation imaging.mp. | 305 |
| 3 | speckle tracking echocardio*.mp. | 2750 |
| 4 | speckle tracking stud*.mp. | 56 |
| 5 | speckle tracking analys*.mp. | 245 |
| 6 | deformation analys*.mp. | 379 |
| 7 | diabetes mellitus/ or diabetes mellitus, type 1/ or diabetes mellitus, type 2/ | 299992 |
| 8 | diabetes mellitus.mp. | 427259 |
| 9 | 3 or 4 or 5 | 2946 |
| 10 | 7 or 8 | 427259 |
| 11 | 1 and 2 and 9 | 10 |
| 12 | 1 or 2 or 6 or 9 | 4330 |
| 13 | 10 and 12 | 160 |
| 14 | (left ventric* or LV or right ventric* or RV or left atri* or LA or right atri* or RA).mp. | 881773 |
| 15 | (function* or dysfunction*).mp. | 4093791 |
| 16 | 14 and 15 | 191576 |
| 17 | strain.mp. | 430336 |
| 18 | 16 and 17 | 8130 |
| 19 | 13 and 18 | 127 |
| 20 | 13 or 19 | 160 |
| 21 | Filter by journal article and human studies | 121 |

**Embase Classic+Embase (1947 to March 27, 2020)**

|  | **Search Statement** | **Results** |
| --- | --- | --- |
| 1 | myocardial strain.mp. | 2398 |
| 2 | deformation imaging.mp. | 683 |
| 3 | speckle tracking echocardio*.mp. | 8331 |
| 4 | speckle tracking stud*.mp. | 171 |
| 5 | speckle tracking analys*.mp. | 860 |
| 6 | deformation analys*.mp. | 689 |
| 7 | diabetes mellitus/ or diabetes mellitus, type 1/ or diabetes mellitus, type 2/ | 659383 |
| 8 | diabetes mellitus.mp. | 954813 |
| 9 | 3 or 4 or 5 | 8984 |
| 10 | 7 or 8 | 954813 |
| 11 | 1 and 2 and 9 | 22 |
| 12 | 1 or 2 or 6 or 9 | 11581 |
| 13 | 10 and 12 | 794 |
| 14 | (left ventric* or LV or right ventric* or RV or left atri* or LA or right atri* or RA).mp. | 1443344 |
| 15 | (function* or dysfunction*).mp. | 5908112 |
| 16 | 14 and 15 | 350926 |
| 17 | strain.mp. | 842307 |
| 18 | 16 and 17 | 20484 |
| 19 | 13 and 18 | 574 |
| 20 | 13 or 19 | 794 |
| 21 | Filter by journal articles and human studies | 330 |

**Cochrane central register of controlled trials (1991 to February 2020)**

| **#** | **Search Statement** | **Results** |
| --- | --- | --- |
| 1 | myocardial strain.mp. | 101 |
| 2 | deformation imaging.mp. | 16 |
| 3 | speckle tracking echocardio*.mp. | 300 |
| 4 | speckle tracking stud*.mp. | 3 |
| 5 | speckle tracking analys*.mp. | 33 |
| 6 | deformation analys*.mp. | 12 |
| 7 | diabetes mellitus/ or diabetes mellitus, type 1/ or diabetes mellitus, type 2/ | 25548 |
| 8 | diabetes mellitus.mp. | 63955 |
| 9 | 3 or 4 or 5 | 324 |
| 10 | 7 or 8 | 63955 |
| 11 | 1 and 2 and 9 | 0 |
| 12 | 1 or 2 or 6 or 9 | 417 |
| 13 | 10 and 12 | 26 |
| 14 | (left ventric* or LV or right ventric* or RV or left atri* or LA or right atri* or RA).mp. | 55341 |
| 15 | (function* or dysfunction*).mp. | 278968 |
| 16 | 14 and 15 | 20338 |
| 17 | strain.mp. | 8265 |
| 18 | 16 and 17 | 803 |
| 19 | 13 and 18 | 19 |
| 20 | 13 or 19 | 26 |
| 21 | Filter by journal articles and human studies | 10 |

**Scopus** **(March 30, 2020):** 40 studies

( TITLE-ABS KEY ( myocardial  AND strain  OR  deformation  AND imaging  OR  speckle  AND tracking  AND stud*  OR  speckle  AND tracking  AND analys*  OR  deformation  AND analys* ) )  AND  ( TITLE-ABS-KEY ( diabetes  AND mellitus  OR  diabetes  AND mellitus  AND type  1  OR  diabetes  AND mellitus  AND type  2 ) )  AND  ( LIMIT TO ( LANGUAGE ,  "English" ) )  AND  ( LIMIT-TO ( DOCTYPE ,  "ar" ) )  AND  ( LIMIT-TO ( SUBJAREA ,  "MEDI" ) )  AND  ( LIMIT-TO ( EXACTKEYWORD ,  "Human" ) )

**Web of Science (March 30, 2020):** 290 studies

( myocardial  AND strain  OR  deformation  AND imaging  OR  speckle  AND tracking  AND stud*  OR  speckle  AND tracking  AND analys*  OR  deformation  AND analys* )  AND ( diabetes  AND mellitus  OR  diabetes  AND mellitus  AND type  1  OR  diabetes  AND mellitus  AND type  2 ) Refined by: LANGUAGES: ( ENGLISH ) AND DOCUMENT TYPES: ( ARTICLE ) AND WEB OF SCIENCE CATEGORIES: ( CARDIAC CARDIOVASCULAR SYSTEMS OR ENDOCRINOLOGY METABOLISM )

**Table S1:** **Definition of study groups of included studies**

| **First Author** | **Year** | **DM group inclusion criteria** | **DM group exclusion criteria** | **Control group definition** |
| --- | --- | --- | --- | --- |
| **Nakai** | 2009 | Normal LVEF with no regional wall motion abnormalities | A history of coronary artery disease, the presence of moderate-to-severe valvular heart disease, and/or significant rhythm disturbances | Healthy control adjusted to the same range of age |
| **NG** | 2009 | (1) type 2 diabetes mellitus diagnosed according to the World Health Organization criteria and treated with sulfonylurea derivatives in stable doses, (2) haemoglobin A1c <8.5%, and (3) blood pressure at rest of <150/85 mm Hg, with or without antihypertensive medication | Presence of myocardial ischemia on a high-dose dobutamine stress echocardiogram, known cardiovascular disease or diabetes-related complications, including proliferative retinopathy, autonomic neuropathy and microalbuminuria | Male control subjects were matched for age, body mass index, and body surface area. All control subjects had normal physical examination and normal echocardiographic findings. The exclusion criteria for the control subjects included a history of diabetes mellitus, smoking, hypertension, and cardiomyopathy |
| **Ernande** | 2011 | Age between 35 and 60 years, no symptoms or history of heart disease, LV ejection fraction (LVEF) > 55%, absence of regional LV wall motion abnormalities assessed by echocardiography, and no myocardial ischemia | Absence of sinus rhythm, coronary and valvular heart diseases, severe renal failure, echocardiographic images unsuitable for quantification, type 1 DM, severely uncontrolled DM (glycosylated haemoglobin > 12%), and uncontrolled blood pressure at rest (defined as systolic blood pressure > 180 mm Hg and/or diastolic blood pressure > 100 mm Hg) | Age-matched subjects with the DM group and without cardiovascular risk factors. Subjects were eligible if they me the following criteria: never smokers, systolic blood pressure < 140 mm Hg and diastolic blood pressure < 85 mm Hg on triplicate measurement, no drug treatments for hypertension, no diabetes, and normal fasting glycemia (glycemia < 110 mg/dL), triglycerides < 150 mg/dL, total cholesterol < 230 mg/dL, low-density lipoprotein cholesterol < 160 mg/dL, high-density lipoprotein cholesterol > 38.5 mg/dL, and serum creatinine < 1.25 mg/dL |
| **Mondillo** | 2011 | Diabetic patients with echocardiographic evidence of normal LA size, defined as LA volume indexed to body surface area < 28 mL/m2 , and no symptoms of suspected cardiac origin | Overt coronary artery disease (defined by at least one of the following: history of effort angina, acute coronary syndromes, or revascularization procedures; evidence of positive exercise stress test results; and segmental wall abnormalities at echocardiography); history of atrial fibrillation, atrial flutter, or other major arrhythmias; mitral regurgitation of higher degree than trivial; aortic regurgitation; any degree of valve stenosis; previous valve surgery; hypertrophic cardiomyopathy; left bundle branch block; pacemaker implantation; heart transplantation; malignant hypertension; uncontrolled diabetes (defined as glycosylated haemoglobin > 7.0%), severe renal failure (defined as estimated creatinine clearance < 30 mL/min); history of chronic obstructive pulmonary disease, abnormal thyroid function, or other significant systemic chronic disease; inadequate acoustic windows; and refusal to participate in the study | Healthy individuals with no histories of any cardiovascular or systemic disease and with normal findings on clinical examination, electrocardiography matching for age was performed using a 1:4 scheme and to within 65 years |
| **D'Andrea** | 2012 | Normotensive patients with non-insulin DM of ≥4 years duration, in sinus rhythm, with normal LV global systolic function (LV ejection fraction ≥50%) and microvascular angina (anginal pain, positive imaging stress test and normal coronary angiography) | Arterial hypertension, coronary artery disease (significant epicardial coronary stenosis), ≥ moderate valvular heart disease, New York Heart Association (NYHA) functional classes II–IV, and inadequate echocardiograms | Age- and sex-matched healthy controls with no cardiovascular, structural or functional abnormalities or received any medication |
| **Kadappu** | 2012 | Diabetic patients with normal LV ejection fraction | ECG or echocardiographic evidence of coronary artery disease, greater than mild mitral or aortic regurgitation or aortic stenosis or any degree of mitral stenosis. had any history of congestive heart failure | Age-matched ‘normal’ subjects, no any RF, no abnormal findings on routine physical examination, were normotensive and had a normal ECG and echocardiogram and were not receiving any cardio-active medications |
| **Conte** | 2013 | Stable asymptomatic diabetes patients | Concomitant moderate-to-severe valve regurgitation or stenosis, depressed LVEF (50%), previous history of CAD (Coronary Artery Disease) or recovery for CHF (Congestive Heart Failure) or other cardiovascular causes. | Healthy volunteers with no medical history of cardiovascular or pulmonary pathologies |
| **Tadic** | 2014 | Normotensive patients untreated type 2 diabetes | Symptoms or signs of cardiovascular disease (arterial hypertension, heart failure, myocardial infarction, significant valvular disease, atrial fibrillation, congenital heart disease), obesity (BMI C 30 kg/m2 ), asthma, chronic obstructive lung disease, neoplastic disease, cirrhosis of the liver, or kidney failure | Similar age and gender controls |
| **Zoroufian** | 2014 | Type 2 DM diagnosed according to the World Health Organization criteria, normal coronary arteries proved by coronary angiography and LV ejection fraction (LVEF) >50%, and no regional wall-motion abnormality | History of hypertension with or without antihypertensive drug consumption, absence of stable sinus rhythm, conduction and rhythm disturbances, abnormal serum liver enzyme levels, endocrine and other than DM systemic disease, renal impairment with serum creatinine level ≥1.5 mg/dL, and valvular or congenital heart disease | Sex-matched control subjects (45.9% males) with LVEF >50% and without diabetes were included. Control subjects had also normal coronary angiography and their exclusion criteria were as same as the patient’s group |
| **Bakirci** | 2015 | Type 2 diabetic patients who were diagnosed at least one year prior | History of coronary artery disease, left ventricular (LV) systolic dysfunction, moderate to severe valvular heart disease, arterial hypertension, AF, pre-excitation syndromes, atrioventricular conduction abnormalities, bundle branch block, previously implanted cardiac pacemakers, anti-arrhythmic medicine use, electrolyte imbalance, thyroid diseases, chronic renal or liver diseases, pulmonary disease, chronic inflammatory disorders, known infectious disease and a history of a hypoglycemic episode in the past month | Sex- and age matched healthy controls |
| **Enomoto** | 2015 | Hospitalized normotensive patients with poorly controlled type 2 diabetes | Coronary artery disease, significant mitral or aortic valvular heart disease, a rhythm other than normal sinus rhythm, or LVEF <50% | Age and sex-matched healthy controls |
| **Jensen** | 2015 | Patients were eligible if they were 18 years or older, attending the outpatient clinic at Steno Diabetes Centre, diagnosed with T1DM, without known heart disease, and willing to participate | Known heart disease was defined as heart failure; coronary artery disease (CAD), including previous myocardial infarction, stable angina, previous percutaneous coronary intervention, or coronary artery bypass surgery; atrial fibrillation or atrial flutter; left bundle branch block; congenital heart disease; and pacemaker or implantable cardioverter defibrillator insertion | Subjects with similar age and sex distribution were included as control subjects if they had no history of CVD in the National Patient Registry; answered “No” to previous CVD on the questionnaire; did not take any cardiovascular medication; did not have diabetes; did not have known kidney disease; did not have known hypertension |
| **Karagov** | 2015 | Patients with the diagnosis of type II DM ((a) a fasting blood glucose of >126 mg/dl measured on two different occasions or (b) patients being on oral antidiabetic drugs and/or insulin) | Established or suspected coronary artery disease (e.g., typical angina pectoris, positive exercise test or myocardium perfusion scintigraphy, segmentary wall motion abnormalities of LV), atrial fibrillation, and poor acoustic windows | Healthy controls |
| **Skali** | 2015 | Patients from ARIC study with known diabetes mellitus or on anti–diabetes mellitus medications (visit 5-HbA1c ≥ 6.5%, visit 5-fasting glucose ≥126 mg/ dL or non-fasting glucose>200 mg/dL) | Non-white or nonblack race, without echocardiographic examination, with prevalent heart disease (HF, CHD, previous myocardial infarction, cardiac pacemaker or defibrillator in place), or valvular disease (moderate or severe, previous valve repair or replacement) | Patients from ARIC study with no known diabetes mellitus at visits 1 to 5 and annual follow-up data, and visit 5-HbA1c < 5.7% and visit 5-fasting glucose level |
| **Tadic** | 2015 | Normotensive patients (blood pressure < 140/90 mm Hg measured on several separate occasions) untreated for type 2 diabetes | Symptoms or signs of cardiovascular disease (arterial hypertension, angina pectoris, heart failure, myocardial infarction, significant valvular disease, atrial fibrillation, congenital heart disease), obesity (body mass index [BMI] >30 kg/m2), asthma, chronic obstructive lung disease, neoplastic disease, cirrhosis of the liver, or kidney failure | Controls of similar age and sex distributions |
| **Tadic** | 2015 | Normotensive patients ((blood pressure < 140/90 mm Hg measured on several separate occasions) untreated for type 2 diabetes | Symptoms or signs of cardiovascular disease (arterial hypertension, heart failure, myocardial infarction, significant valvular disease, atrial fibrillation, congenital heart disease), obesity (BMI ≥ 30 kg/m2 ), asthma, chronic obstructive lung disease, neoplastic disease, cirrhosis of the liver, or kidney failure | Controls of similar age and sex distributions |
| **Abdel-Salem** | 2016 | Patients with age < 40 years, type 1 DM diagnosed for >5 years and LVEF>50% | Hypertension, history or ECG suggestive of CAD, symptoms or signs of CHF, RWMA in >2 segments, LVH, LV pacing, valvular or congenital heart disease, known cerebrovascular or peripheral vascular disease | Age and gender-matched control, without HTN, dyslipidaemia, familial history of CAD and non-took regular medications |
| **Bakhum** | 2016 | Asymptomatic T1DM patients without known cardiovascular disease, have never smoked and with an LV ejection fraction >55% by biplane Simpson’s method | Hypertension defined as blood pressure > 140/90, CAD, moderate to severe valvular heart disease, congenital heart disease and atrial fibrillation or other severe arrhythmias | Age and sex-matched, healthy non-diabetic, normotensive and never smoker subjects with no other comorbid conditions |
| **Jedrzejewska** | 2016 | Diabetic patients with diabetes duration ≥9 years, left ventricular ejection fraction (LVEF) .55%, normal resting electrocardiogram (ECG), and normal electrocardiographic exercise stress test within 3 weeks before inclusion | Hypertension, type 2 DM, history or symptoms of CAD, valvular heart disease, rhythm disturbances, cardiomyopathy, congenital heart disease, symptomatic heart failure, pulmonary disease, endocrine disease, and renal failure (defined as creatinine clearance <60 mL/min) | Healthy subjects in the same range of age with inclusion criteria of age 18 – 43 years, normal physical examination, BP<140/85 mmHg measured twice at one visit, no history of DM or cardiovascular disease, no treatment for hypertension or hypercholesterolaemia, fasting blood glucose <5.6 mmol/L, total cholesterol <6.5 mmol/L (250 mg/dL), low-density lipoprotein cholesterol (LDL) <4.1 mmol/L (160 mg/dL), high-density lipoprotein cholesterol (HDL) >1 mmol/L (40 mg/dL), and triglycerides <1.7 mmol/L (150 mg/dL). |
| **Jorgensen** | 2016 | Representative sample of T2D patients was included in the Thousand&2 echocardiographic study | Patients with known coronary heart disease, atrial fibrillation during the echocardiographic examination and presence of more than moderate valve disorder | Age, sex and SBP-matched persons from the Copenhagen City Heart Study |
| **Loncarevic** | 2016 | Asymptomatic DM patient without HTA and CAD (no previous myocardial infarction, no angina pectoris) and with negative stress echocardiography | Unstable angina pectoris, pervious myocardial infarction, uncontrolled HTA, congenital heart diseases, primary hypertrophic and dilated cardiomyopathy, significant heart valve disease, left bundle branch block, atrial fibrillation, severe form of ventricular arrhythmias (Lown class IV and V), anaemia, malignancy, severe obstructive pulmonary disease, disorders of thyroid function, myocarditis and deformities of the chest that technically limit echocardiographic examination | Individuals similar in age and gender as DM patients and with normal echo studies, without DM, cardiology or other major health problems |
| **Mochizuki** | 2016 | Consecutive DM patients | (1) history of coronary artery disease (CAD); (2) LVEF 180/100mmHg; (6) more than moderate valvular heart disease; (7) AF or flutter; and (8) left bundle branch block | Normal control group consisting of age-, sex- and LVEF-matched subjects without a history of hypertension, DM, malignant disease or other cardiovascular disease and none of whom showed abnormal ECG findings |
| **Tadic** | 2016 | Patients with type 2 diabetes | Symptoms or signs of cardiovascular diseases (heart failure, myocardial infarction, significant valve disease, atrial fibrillation, congenital heart disease), obesity (BMI >30 kg/m2 ), neoplastic disease, cirrhosis of the liver or kidney failure | Control subjects |
| **Kishi** | 2017 | Participants of CARDIA (Coronary Artery Risk Development in Young Adults) study with DM diagnosed at year 15 or later | Not attending the year 25 (2010 to 2011) examination, did not have an echocardiogram, women who were pregnant at the year 0 to 25 examination, and whom covariate data were missing | Participants of CARDIA (Coronary Artery Risk Development in Young Adults) study with no DM and no IGT/IFG |
| **Suto** | 2017 | Asymptomatic T2DM patients admitted to Kobe University Hospital | (1) previous or current history of HF; (2) previous history or suspicion of coronary artery disease; (3) LVEF    180/100  mmHg; (7) more than moderate valvular heart disease; and (8) atrial fibrillation | Control group randomly taken from the database by the observers who have no involvement in echocardiographic analysis to have a similar age, gender and LVEF distribution, and consisting of subjects without a history of DM or other cardiovascular disease |
| **Tadic** | 2017 | Normotensive uncomplicated patients with type 2 diabetes | Antihypertensive treatment, heart failure, coronary artery disease, previous cerebrovascular events, atrial fibrillation, congenital heart disease, more than mild valve heart disease, neoplastic disease, cirrhosis of the liver or kidney failure | Normotensive controls free of cardiovascular diseases |
| **Tadic** | 2017 | Normotensive uncomplicated patients with type 2 diabetes | Arterial hypertension, antihypertensive treatment, heart failure, coronary artery disease, previous cerebrovascular events, atrial fibrillation, congenital heart disease, more than mild valve heart disease, obesity (BMI ≥ 35 kg/m2 ), neoplastic disease, cirrhosis of the liver or kidney failure | Normotensive subjects free of cardiovascular diseases |
| **Vukomanovic** | 2017 | Consecutive normotensive uncomplicated patients with type 2 diabetes | Antihypertensive treatment, heart failure, coronary artery disease, previous cerebrovascular events, atrial fibrillation, congenital heart disease, more than mild valvular heart disease, obesity (BMI ≥35 kg/m2 ), neoplastic disease, cirrhosis of the liver or kidney failure, >10% premature supraventricular or ventricular contractions | Normotensive subjects free of cardiovascular diseases |
| **Ahmed** | 2018 | Patients with TIDM with no history of cardiac disease | History of documented cardiac disease (myocardial infarction [MI], episodes of ischemia, heart failure [HF]), inducible ischemia evaluated by exercise ECG or stress echocardiography performed within 1 year of the study, rheumatic heart disease, congenital heart disease, hypertension, arrhythmias, chronic pulmonary disease, smoking or ex-smoking | Healthy subjects matched for age and BMI with low risk for CAD |
| **Jorgensen** | 2018 | Patients with T2D from The Thousand&2 study without any metabolic risk factor (elevated SBP, elevated BMI, reduced HDL, elevated TG, albuminuria, elevated HbA1c) | Patients with atrial fibrillation during the echocardiographic examination, more than moderate valve disease, and/or previous heart valve surgery, patients with incomplete information on BMI, systolic blood pressure, HbA1c, HDL cholesterol, triglyceride levels, or albuminuria status | Randomly matched 4:1 on age, sex, and systolic blood pressure with people from the Copenhagen City Heart Study without diabetes mellitus, known heart disease, or any of the metabolic risk factors |
| **Lin** | 2018 | Known diabetes mellitus or on antidiabetic medications | History of HF, coronary heart disease, myocardial infarction, cardiac pacemaker or defibrillator in place, or valvular heart disease | Healthy control with no known diabetes mellitus with fasting glucose level <100mg/ml and glycosylated haemoglobin (HbA1c) <5.7% |
| **NG** | 2018 | Known type 2 diabetes, which was diagnosed according to World Health Organization criteria | Age < 18 years, rhythm other than sinus rhythm, LV ejection fraction (LVEF) < 50%, moderate or severe valvular stenosis or regurgitation, and congenital heart disease | Subjects without diabetes of similar age, gender, and BMI |
| **Philouze** | 2018 | Known type 2 diabetes | Severe obesity, insulin therapy, LV ejection fraction < 55%, known cardiovascular diseases, and T2DM-related complications, including moderate to severe autonomic neuropathy, proliferative retinopathy, nephropathy and poor echogenicity | Healthy control subjects of similar age and sex |
| **Ringle** | 2018 | Patients aged older than 18 years with isolated type 1 diabetes | Recent diagnosis of diabetes (<1year), documented cardiac disease, diabetic nephropathy, cardiovascular risk factors (hypertension, hypercholesterolemia, active smoking, obesity, age over 60 years) | Age- and gender-matched healthy subjects that met the following criteria: no cardiovascular risk factors, no personal history of heart disease, no clinical history of chronic disease or chronic medication and normal transthoracic echocardiography |
| **Stevanovic** | 2018 | Asymptomatic normotensive type-2 diabetic patients | Clinical manifestations of diabetic complications, reduced left ventricular ejection fraction (EF), presence of myocardial ischemia | Healthy subjects of matching age and sex |
| **Tadic** | 2018 | Patients with DM | Symptoms or signs of cardiovascular disease (heart failure, myocardial infarction, significant valvular disease, atrial fibrillation, congenital heart disease), obesity (body mass index [BMI] ≥ 30 kg/m2), neoplastic disease, cirrhosis of the liver or kidney failure | Control subjects |
| **Berceanu** | 2019 | Patients with type 1 DM and age<40 years and a good image quality | Presence of moderate-to-severe valvulopathies, signs and symptoms of coronary artery disease and/or significant rhythm disturbances | Healthy subjects |
| **Bogdanovic** | 2019 | Patients with diabetes who had optimal metabolic control (HbA1c≤7.0%) with fasting plasma glucose | Patients younger than 18 or older than 65 years of age, with unstable angina pectoris, AMI or history of coronary interventions, heart failure, atrial fibrillation or irregular rhythm on ECG, malignant diseases, obstructive pulmonary disease, hepatic or renal failure (eGFR≤60  ml/min/1.73  m2 ), acute or chronic infections, new uncontrolled hypertension (blood pressure higher than 180/110 mmHg) requiring treatment | Healthy volunteers |
| **Cameli** | 2019 | Asymptomatic patients with type 2 DM and normal LA volume, absent LV dilation and preserved LVEF | Malignant hypertension; uncontrolled DM (glycosylated hemoglobin≥7.0%), manifest coronary artery disease, mitral regurgitation of higher degree than mild; aortic regurgitation; any degree of valve stenosis; previous valve surgery; history of atrial fibrillation, atrial flutter, or other major arrhythmias; hypertrophic cardiomyopathy; left bundle branch block; pacemaker implantation; heart transplantation; severe renal failure, chronic obstructive pulmonary disease; abnormal thyroid function; other significant systemic chronic disease; inadequate acoustic windows; and refusal to participate in the study | Age-matched healthy individuals with no history of any cardiovascular or systemic disease, normal findings on clinical examination, electrocardiography, and echocardiography |
| **Haley** | 2020 | Youth with T2DM who islet cell antibody were negative, had no evidence of other types of diabetes, and did not require insulin in the basal state to prevent diabetic ketoacidosis | Pregnant females and subjects with pre-existing cardiac diseases | Lean control (BMI <85^th^ percentile) matched by age, race, and gender |
| **Roberts** | 2020 | Diabetic patients with age 18-70 years and the ability to perform moderate-intensity exercise | Known artery disease, resting LV systolic dysfunction (LV ejection fraction [LVEF] < 40%), significant nephropathy (estimated glomerular filtration rate < 30 mL/m/minute2 ), and chronic obstructive airways disease | Age- and sex-matched controls |

| **First Author** | **Year** | **Ultrasound system** | **Software** | **Software Version** | **Vendor** |
| --- | --- | --- | --- | --- | --- |
| **Nakai** | 2009 | Vivid 7 | EchoPAC | 6 | GE |
| **NG** | 2009 | Vivid 7 | EchoPAC | 7 | GE |
| **Ernande** | 2011 | Vivid 7 | EchoPAC | NA | GE |
| **Mondillo** | 2011 | Vivid 7 | EchoPAC | NA | GE |
| **D'Andrea** | 2012 | Vivid 7 | AFI | NA | GE |
| **Kadappu** | 2012 | Vivid 7 | EchoPAC | NA | GE |
| **Conte** | 2013 | Vivid 7 | EchoPAC | NA | GE |
| **Tadic** | 2014 | Vivid 7 | EchoPAC | 110.1.2 | GE |
| **Zoroufian** | 2014 | EKO 7 | EKO 7 | NA | Samsung Medison |
| **Bakirci** | 2015 | Vivid S5 | EchoPAC | NA | GE |
| **Enomoto** | 2015 | Artida | 2D Wall Motion Tracking | NA | Toshiba |
| **Jensen** | 2015 | Vivid 7 | EchoPAC | BT11 | GE |
| **Karagov** | 2015 | Vivid 7 | EchoPAC | 7.1,2 | GE |
| **Skali** | 2015 | IE33 | TomTec | NA | Philips |
| **Tadic** | 2015 | Vivid 7 | EchoPAC | 110.1.2 | GE |
| **Tadic** | 2015 | Vivid 7 | EchoPAC | 110.1.2 | GE |
| **Abdel-Salem** | 2016 | Vivid 7 | EchoPAC | NA | GE |
| **Bakhum** | 2016 | Mylab 60 Xvision | MyLab 60 Xvision | NA | Esaote |
| **Jedrzejewska** | 2016 | Vivid 7 | EchoPAC | 8 | GE |
| **Jorgensen** | 2016 | Vivid 7/E9 | EchoPAC | BT13 | GE |
| **Loncarevic** | 2016 | MyLab 30 | X-Strain | NA | Esaote |
| **Mochizuki** | 2016 | Vivid E9 | EchoPAC | 113 | GE |
| **Tadic** | 2016 | Vivid 7 | EchoPAC | 8 | GE |
| **Kishi** | 2017 | Artida | 2D Wall Motion Tracking | NA | Toshiba |
| **Suto** | 2017 | Vivid E9 | EchoPAC | 113 | GE |
| **Tadic** | 2017 | Vivid 7 | EchoPAC | 2.1 | GE |
| **Tadic** | 2017 | Vivid 7 | EchoPAC | 201 | GE |
| **Vukomanovic** | 2017 | Vivid 7 | EchoPAC | 201 | GE |
| **Ahmed** | 2018 | Vivid 7 | EchoPAC | 110.1.2 | GE |
| **Jorgensen** | 2018 | Vivid E9 | EchoPAC | NA | GE |
| **Lin** | 2018 | Vivid i | EchoPAC | 10.8 | GE |
| **NG** | 2018 | Vivid 7 | EchoPAC | 108.1.5 | GE |
| **Philouze** | 2018 | Vivid E95 | EchoPAC | 201 | GE |
| **Ringle** | 2018 | IE33 | TomTec | NA | Philips |
| **Stevanovic** | 2018 | Vivid 7 | EchoPAC | NA | GE |
| **Tadic** | 2018 | Vivid 7 | EchoPAC | 201 | GE |
| **Berceanu** | 2019 | Vivid S6 | EchoPAC | 110 | GE |
| **Bogdanovic** | 2019 | Vivid E9 | EchoPAC | 113 | GE |
| **Cameli** | 2019 | Vivid E9 | EchoPAC | NA | GE |
| **Haley** | 2020 | Vivid 5/7 | EchoPAC | NA | GE |
| **Roberts** | 2020 | Vivid E9 | EchoPAC | 113 | GE |

**Table S2: Ultrasound system Information of included studies**

**Table S3: Summary of included articles**

| **First Author** | **Year** | **Rest SBP Mean±SD (DM)** | **Rest SBP Mean± SD (Control)** | **Rest DBP Mean± SD (DM)** | **Rest DBP Mean± SD (Control)** | **Rest HR Mean±SD (DM)** | **Rest HR Mean± SD (Control)** | **DM Duration Mean** | **DLP % (DM)** | **DLP % (Control)** | **Statin use**  **% (DM)** | **Statin use %**  **(Control)** | **Insulin use (%)** | **Oral Antidiabetic use %** | **Metformin use (%)** | **Sulfonylurea use (%)** | **Smoking %**  **(DM)** | **Smoking % (Control)** | **HbA1C Mean± SD (DM)** | **HbA1C Mean±SD (Control)** |
| --- | --- | --- | --- | --- | --- | --- | --- | --- | --- | --- | --- | --- | --- | --- | --- | --- | --- | --- | --- | --- |
| **Nakai** | 2009 |  |  |  |  |  |  | 8.7±8.4 | 45 | 32 |  |  | 52 |  |  | 38 | 48 | 24 | 8.1±2.3 |  |
| **NG** | 2009 | 137±11 | 128±13 |  |  | 72±10 | 66±8 |  |  |  |  |  |  |  |  |  |  |  | 6.4±0.7 |  |
| **Ernande** | 2011 | 128±14 | 120±9 | 77±10 | 74±6 | 75±12 | 63±8 |  |  |  | 46 | 0 | 42 |  | 74 | 47 |  |  |  |  |
| **D'Andrea** | 2012 | 127.4±8.7 | 123.4±7.2 | 72±8.7 | 70.8±10.9 | 78.1±7.6 | 76.9±10.2 | 5.6±4.3 |  |  |  |  |  |  | 43 | 31.5 |  |  | 6.9±1.1 |  |
| **Conte** | 2013 | 143±15.9 | 120.5±9.8 | 81.5±9.2 | 78.8±5.6 | 76.5±12.6 | 68.9±10.6 | 9.1±9.5 |  |  |  |  |  |  |  |  |  |  | 7.3±1 |  |
| **Zoroufian** | 2014 | 116±7.9 | 116±6.2 | 74±6.4 | 77±2.7 | 74±12 | 70±10 | 7.7±7.7 | 33.3 | 5.4 | 33.3 | 5.4 | 17.9 | 84.6 |  |  | 2.6 | 5.4 | 7.9±1.6 |  |
| **Enomoto** | 2015 | 112±16 | 113±15 | 68±10 | 65±13 | 68±12 | 65±11 | 7.7±7.5 |  |  |  |  | 55 | 31 |  |  |  |  | 10.3±2.6 |  |
| **Jensen** | 2015 | 133±17 | 130±12 | 74±10 | 78±8 |  |  | 26.1±15.7 |  |  | 43.2 | 0 |  |  |  |  |  |  | 8.2±1.3 |  |
| **Karagov** | 2015 | 133±14.7 | 134±25.1 | 81±1.5 | 80±19 |  |  |  |  |  |  |  | 21 | 94 |  |  | 24 | 33 |  |  |
| **Skali** | 2015 | 129 | 128 | 66 | 67 | 63 | 60 |  | 66.3 | 37.6 |  |  |  |  |  |  | 64.9 | 65.5 | 6.4 | 5.4 |
| **Tadic** | 2015 | 127±12 | 122±11 | 76±8 | 73±8 | 74±9 | 74±7 |  |  |  |  |  |  |  |  |  | 39 | 26 | 8.1±1.4 | 4.6±0.8 |
| **Tadic** | 2015 | 128±11 | 124±13 | 77±7 | 75±8 | 71±8 | 73±7 |  |  |  |  |  |  |  |  |  |  |  | 7.4±0.7 | 4.6±0.5 |
| **Abdel-Salem** | 2016 | 117±14 | 114±12 | 71±12 | 71±10 | 89±13 | 73±10 |  |  |  |  |  |  |  |  |  |  |  | 7.4±1.7 | 4.9±0.5 |
| **Bakhum** | 2016 | 120.5±15.7 | 120.23±7.2 | 77.3±4.9 | 76.8±5.6 |  |  |  |  |  |  |  |  |  |  |  |  |  |  |  |
| **Jedrzejewska** | 2016 | 122±12.1 | 122±10.5 | 71±7.7 | 71±7.7 | 72±10 | 64±8 | 19.6±6.1 |  |  |  |  |  |  |  |  | 20 | 12 | 7.9±1.6 |  |
| **Jorgensen** | 2016 | 136±17.3 | 133±17.4 | 80±0.9 | 78±0.9 |  |  | 11 |  |  | 77.3 | 15 | 45.5 |  | 74.3 | 15.3 |  |  |  |  |
| **Loncarevic** | 2016 |  |  |  |  |  |  | 7.7±4.9 | 50 | 18.8 |  |  |  |  |  |  |  |  | 6.95±0.85 |  |
| **Mochizuki** | 2016 | 121±16 | 120±15 |  |  | 68±12 | 65±10 | 10±9 | 55 | 0 | 37 |  | 64 |  | 42 | 15 |  |  | 8.6±2.2 |  |
| **Kishi** | 2017 | 124.5±17.4 | 115.8±14.6 | 78.1±10.6 | 72.1±10.7 | 71±5.6 | 65±4.9 |  |  |  |  |  |  |  |  |  | 43.5 | 37.1 | 7.27±1.8 | 5.38±0.4 |
| **Suto** | 2017 | 129±20 | 122±14 | 74±12 | 72±11 | 75±12 | 67±11 |  | 62 | ? | 47.6 |  | 50.3 |  | 54.5 | 24.8 |  |  |  |  |
| **Vukomanovic** | 2017 | 131±9 | 129±10 | 82±7 | 81±7 |  |  | 5±3 |  |  | 44 | 0 |  |  |  |  |  |  | 7.3±1.5 | 5.2±0.4 |
| **Jorgensen** | 2018 | 126±10 | 123±9 | 77±8 | 76±10 |  |  | 7 | 0 | 0 | 86 | 10 | 21.1 |  | 71.9 | 10.5 |  |  | 6.1±0.4 |  |
| **Lin** | 2018 | 131.4±19 | 117.8±15 | 78.7±10.8 | 73.9±10 | 75.6±12.8 | 73.3±11.1 |  | 25.9 | 5.7 |  |  |  |  |  |  | 10.7 | 9.7 | 7.1±1.4 | 5.4±0.2 |
| **NG** | 2018 | 139±20 | 136±24 | 81±11 | 82±12 | 74±13 | 71±13 |  | 51 | 14.2 |  |  |  |  |  |  |  |  | 7.3±1.5 |  |
| **Philouze** | 2018 |  |  |  |  |  |  | 9.5±6.9 |  |  | 30 | 14 |  |  |  |  | 39 | 34 | 7.2±1.1 | 5.4±0.3 |
| **Ringle** | 2018 | 121±12 | 122±9 | 74±9 | 74±6 |  |  | 21±12 | 0 | 0 |  |  |  |  |  |  |  |  |  |  |
| **Stevanovic** | 2018 | 124±7.6 | 121±7.2 | 78±4 | 77±4.6 | 69±9 | 68±7 |  |  |  |  |  |  |  |  |  | 53.7 | 43.9 | 8.2±1.8 | 5.6±1.1 |
| **Tadic** | 2018 | 131±8 | 127±9 | 78±8 | 76±7 | 70±7 | 68±6 | 3 |  |  |  |  | 22.9 |  |  |  |  |  | 7.3±1 | 4.9±0.6 |
| **Bogdanovic** | 2019 |  |  |  |  |  |  | 8.4±1.2 |  |  |  |  | 60 | 40 |  |  |  |  | 6.1±0.1 | 5±0.1 |
| **Cameli** | 2019 | 130±15 | 125±13.3 | 79±8.5 | 80±9 | 70±9 | 74±11 |  | 50 | 35 | 30.8 | 11.7 | 34.6 | 51.9 |  |  | 23.1 | 23.3 |  |  |
| **Haley** | 2020 | 121±15.4 | 111±11.4 | 76.3±10.7 | 69.3±8.6 | 76.4±13.4 | 64.5±10 |  |  |  |  |  |  |  |  |  |  |  | 8.7±3.1 | 4.9±0.5 |
| **Roberts** | 2020 | 125±11 | 121±13 | 76±5 | 76±9 |  |  |  |  |  |  |  |  |  |  |  |  |  | 7.8±1.3 | 5.2±0.2 |

**Table S4:** **Meta-regression results in control groups**

| **Variable** | | **DM type**  **(II vs I)** | **Age, per 1 year** | **%female, per 1%** | **SBP, per 1 mmHg** | **DBP, per 1 mmHg** | **HR, per 1 pm** | **%HTN, per 1%** | **BMI, per 1 kg/m2** | **HbA1C, per 1%** |
| --- | --- | --- | --- | --- | --- | --- | --- | --- | --- | --- |
| **LV GLS** | **N** | 32 | 32 | 32 | 28 | 26 | 20 | 27 | 31 | 13 |
|  | **β [95% CI]** | -0.56 [-2.7, 1.6] | -0.04 [-0.1, 0] | 0.01 [-0.1, 0.1] | 0 [-0.2, 0.2] | 0.04 [-0.2, 0.3] | 0.07 [-0.2, 0.3] | -0.04 [-0.1, 0] | -0.2 [-0.5, 0.1] | -0.56 [-4.9, 3.8] |
|  | ***P-*value** | 0.61 | 0.27 | 0.85 | 0.97 | 0.75 | 0.56 | 0.24 | 0.24 | 0.8 |
| **LV GCS** | **N** | 14 | 14 | 14 | 12 | 11 | 8 | 12 | 13 | 6 |
|  | **β [95% CI]** | -3.81 [-8.5, 0.9] | 0.02 [-0.1, 0.2] | -0.04 [-0.1, 0] | **0.37 [0, 0.7]** | 0.37 [-0.2, 1] | 0.36 [-0.3, 1] | **-0.26 [-0.4,-0.1]** | -0.49 [-1.4, 0.4] | -2.60 [-14, 8.8] |
|  | ***P-*value** | 0.11 | 0.93 | 0.37 | **0.04** | 0.23 | 0.27 | **0.0009** | 0.27 | 0.65 |
| **LV GRS** | **N** | 9 | 9 | 9 | 8 | 7 | 6 |  | 8 | 3 |
|  | **β [95% CI]** | 3.61 [-6.6, 13.8] | 0.21 [-0.3, 0.5] | 0.04 [-0.2, 0.3] | -1.05 [-2.6, 0.2] | -0.53 [-2.2, 1.2] | -0.53 [-1.9, 0.8] |  | -1.21 [-4.9, 2.5] | **-9.67 [-18.1, -1.3]** |
|  | ***P-*value** | 0.49 | 0.55 | 0.73 | 0.09 | 0.54 | 0.43 |  | 0.52 | **0.02** |
| **LV LSSR** | **N** | 13 | 13 | 13 | 11 | 10 | 7 | 11 | 13 | 5 |
|  | **β [95% CI]** | -0.07 [-0.3, 0.1] | 0 [0, 0] | 0 [0, 0] | 0 [0, 0] | -0.01 [0, 0] | **0.05 [0, 0.1]** | 0 [0, 0] | -0.02 [-0.1, 0.1] | -0.33 [-1.2, 0.6] |
|  | ***P-*value** | 0.53 | 0.85 | 0.27 | 0.95 | 0.58 | **0.02** | 0.6 | 0.68 | 0.47 |
| **RV GLS** | **N** | 7 | 7 | 7 | 7 | 7 | 4 | 6 | 7 | 5 |
|  | **β [95% CI]** | 0.68 [-4.7, 6.1] | 0.03 [-0.2, 0.2] | 0.01 [-0.2, 0.2] | 0.27 [-0.3, 0.8] | -0.09 [-0.9, 0.7] | **-0.45 [-0.9, 0]** | -0.48 [-1.7, 0.8] | -0.62 [-2.4, 1.1] | **-3.99 [-5.5, -2.5]** |
|  | ***P-*value** | 0.81 | 0.81 | 0.94 | 0.34 | 0.83 | **0.03** | 0.45 | 0.48 | **<0.0001** |
| **LA reservoir strain** | **N** |  | 7 | 6 | 6 | 5 | 5 |  | 6 |  |
|  | **β [95% CI]** |  | **0.39 [-0.1, 0.7]** | 0.18 [0, 0.4] | 0.29 [-0.2, 0.7] | 0.33 [-0.2, 0.9] | -0.05 [-0.7, 0.6] |  | 0.6 [-0.8, 2] |  |
|  | ***P-*value** |  | **0.02** | 0.06 | 0.2 | 0.23 | 0.88 |  | 0.39 |  |

**Table S5: Strain values with GE vendor only**

| **Strain variable** | **Studies (n)** | **DM (n)** | **Control (n)** | **Mean [95% CI] in DM** | **Mean [95% CI] in Control** | **MD [95% CI]**  **Random Effects model** | **Standardized MD [95% CI]** |
| --- | --- | --- | --- | --- | --- | --- | --- |
| **LVGLS** | 25 | 4115 | 3305 | 18.2 [17.5, 18.9] | 20.2 [19.4, 21.0] | -2.03 [-2.62, -1.44] | -0.9 [-1.1, -0.6] |
| **LVGCS** | 10 | 1062 | 1928 | 20.0 [18.3, 21.6] | 21.0 [19.4, 22.6] | -1.09 [-1.80, -0.38] | -0.4 [-0.6, -0.1] |
| **LVGRS** | 8 | 463 | 374 | 43.5 [40.7, 46.3] | 48.2 [44.6, 51.8] | -4.43 [-5.92, -2.94] | -0.4 [-0.5, -0.3] |
| **LVSR** | 9 | 794 | 751 | 1.0 [0.9, 1.1] | 1.1 [1.0, 1.2] | -0.07 [-0.11, -0.03] | -0.4 [-0.5, -0.2] |
| **LA reservoir strain** | 7 | 543 | 428 | 28.0 [24.4, 31.6] | 36.5 [34.0, 39.0] | -8.42 [-11.6, 5.25] | -1.2 [-1.5, -0.9] |
| **RVGLS** | 7 | 341 | 311 | 23.8 [20.1, 27.4] | 26.0 [23.4, 28.6] | -2.38 [-4.67, -0.09] | -1.1 [-2.3, 0.1] |

**Figure S1: Forest plots for LVGCS**


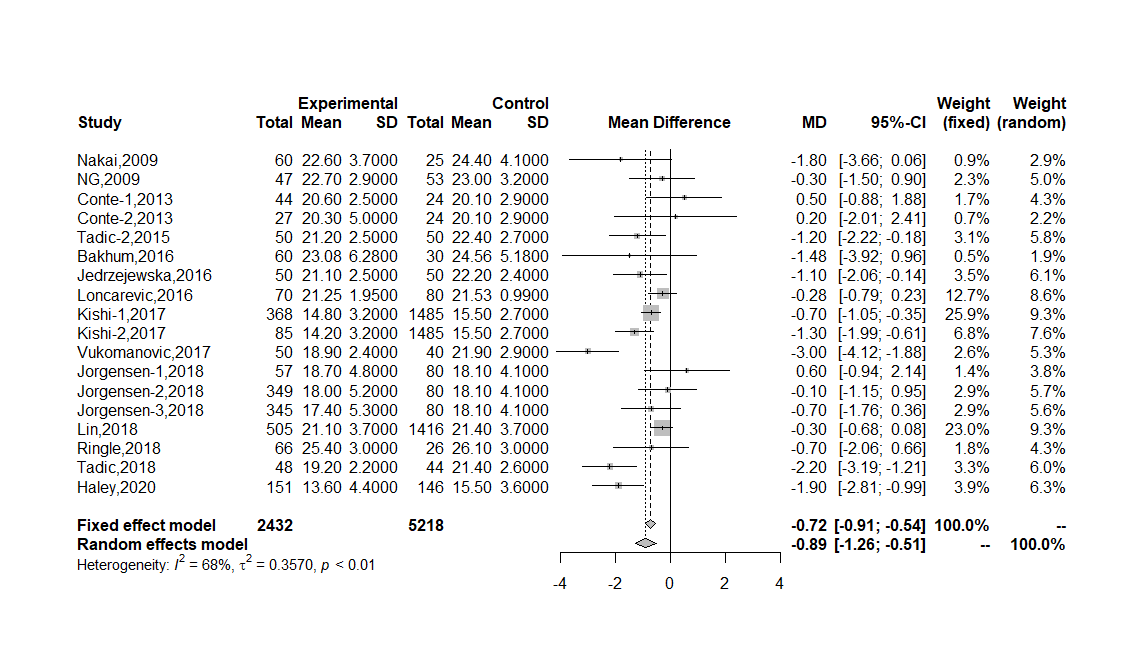


**Figure S2: Forest plots for LVGRS**


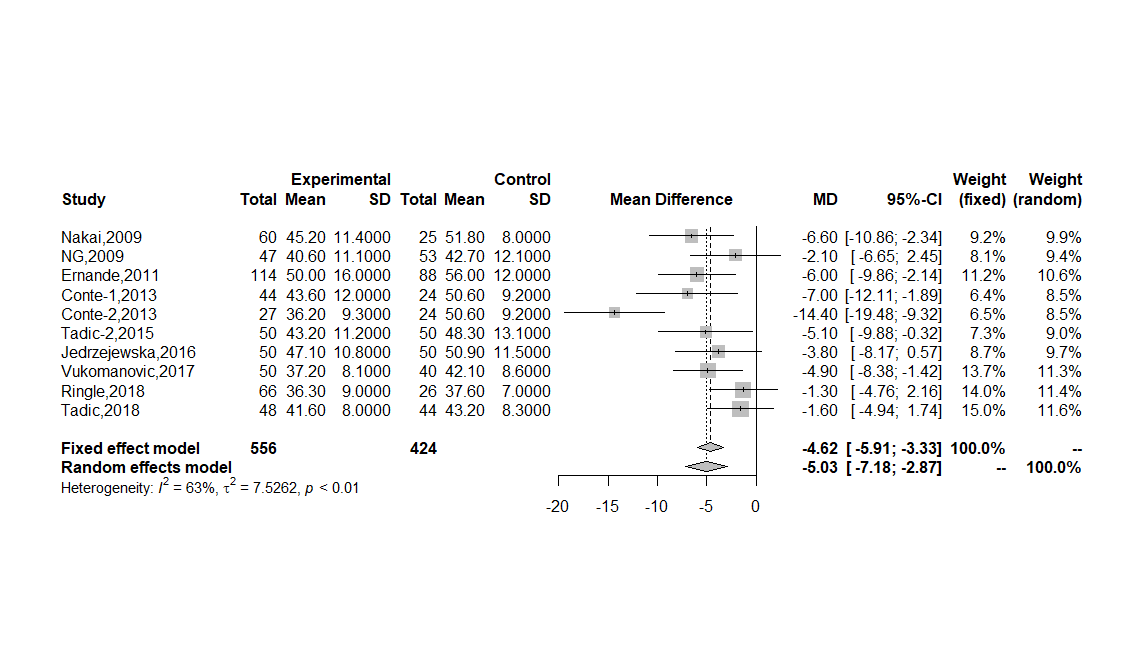


**Figure S3: Forest plots for LVSR**


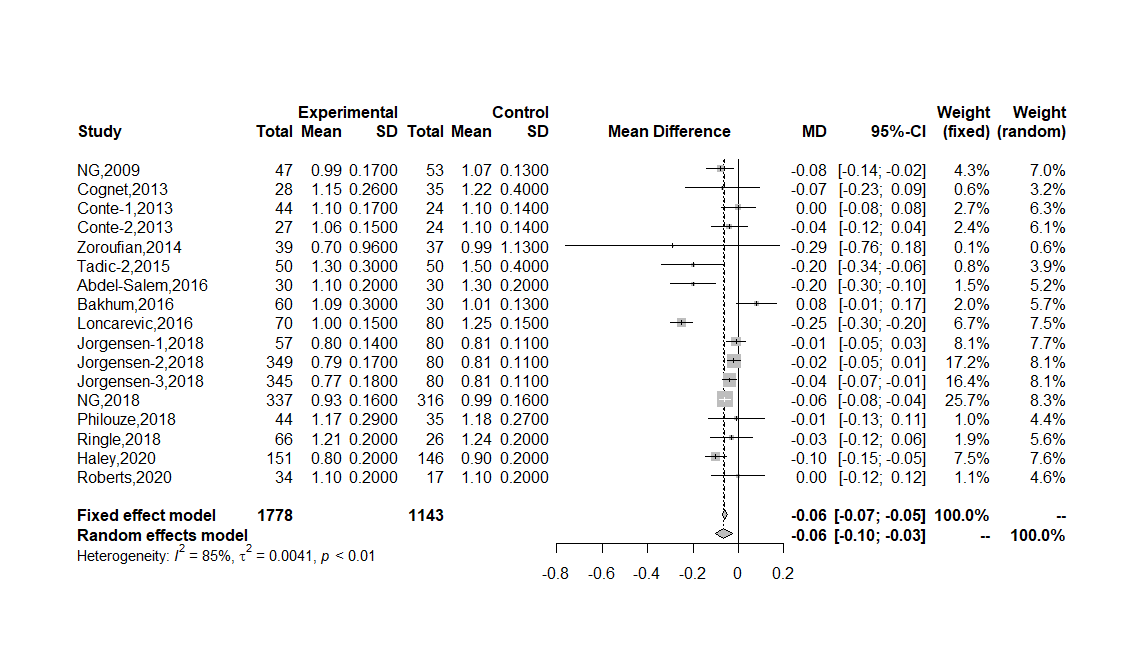


**Figure S4: Funnel plots with Trim and Fill plots for LVGLS**


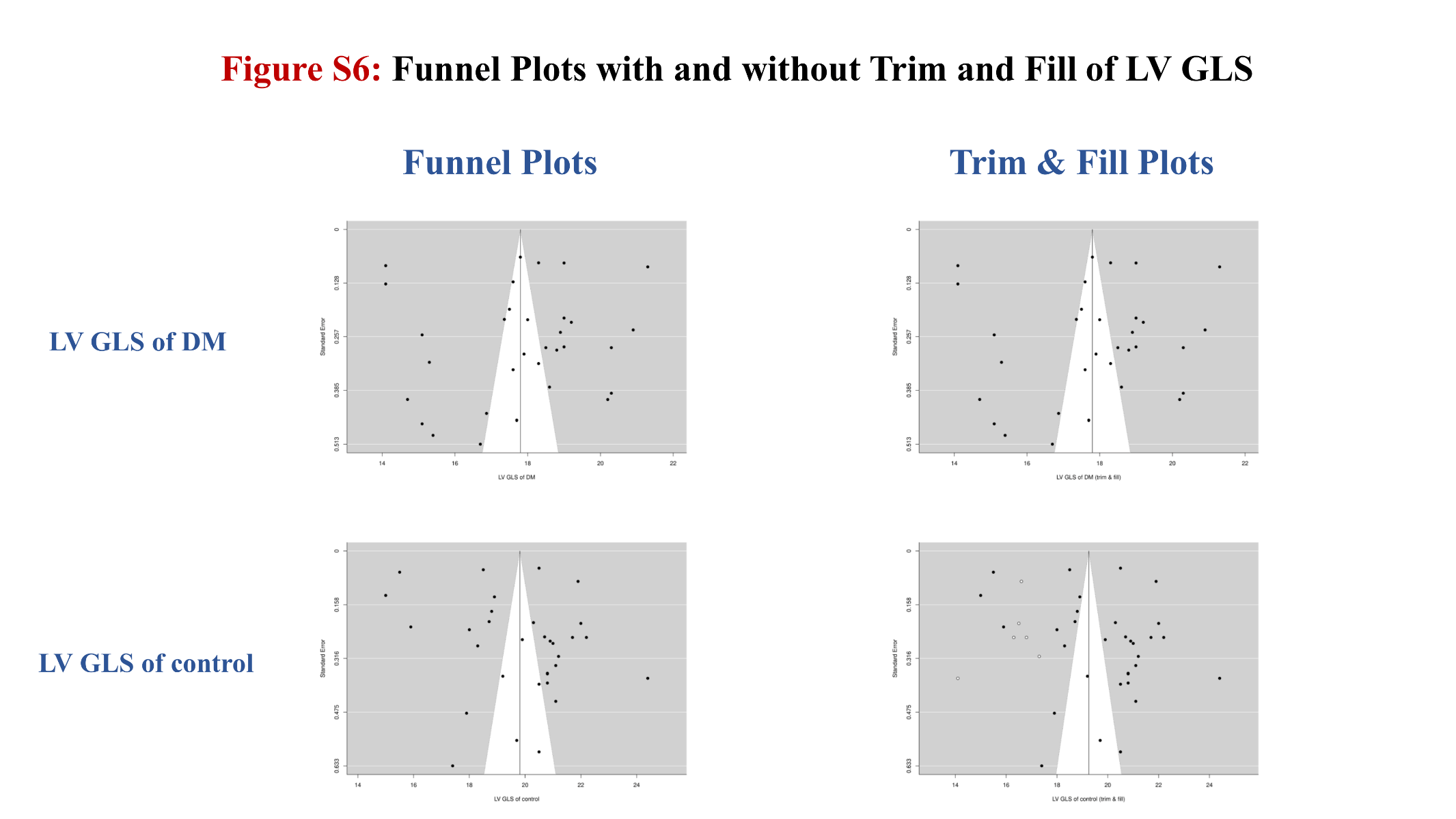


**Figure S5: Funnel plots with Trim and Fill plots for LVGCS**


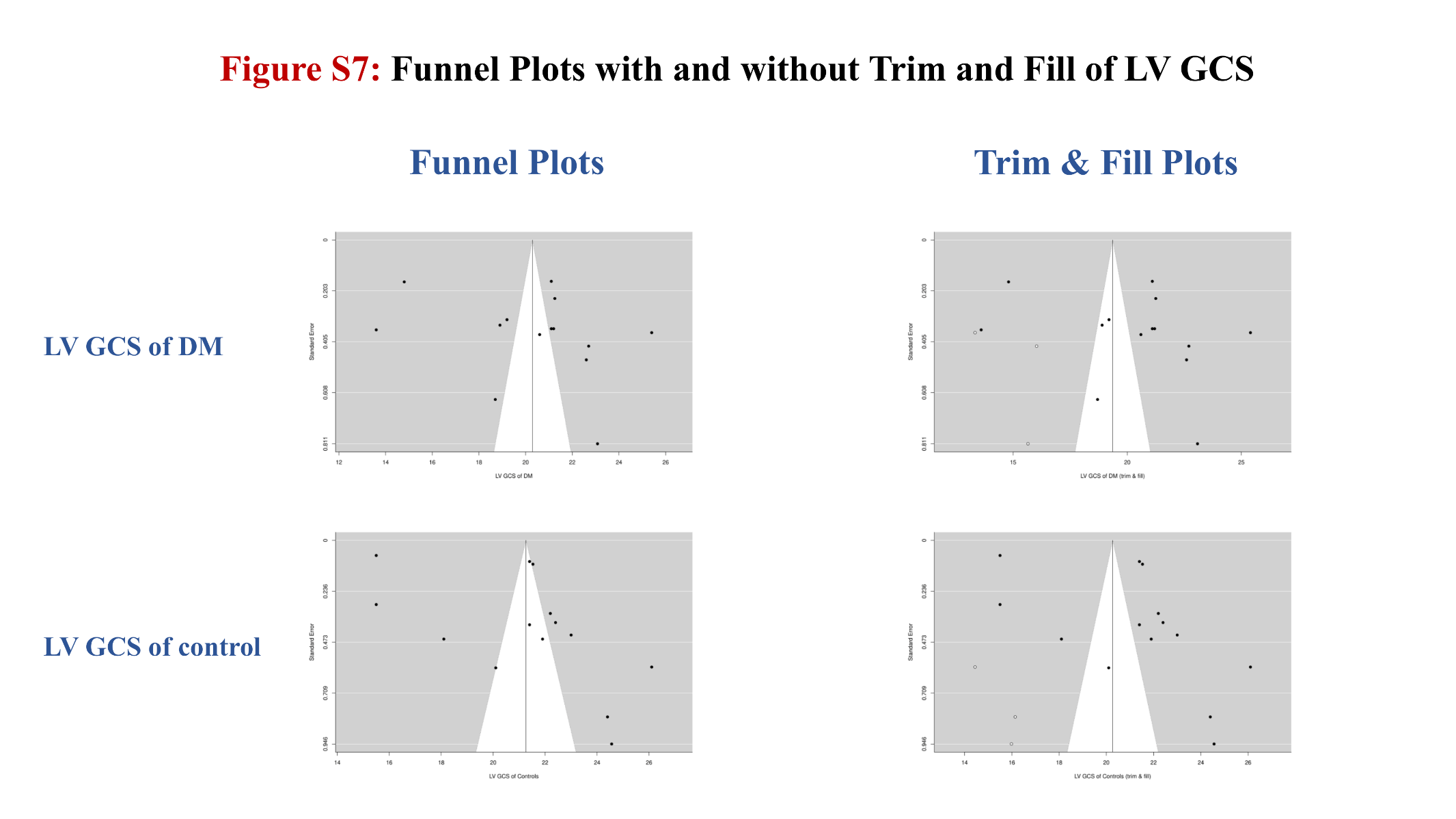


**Figure S6: Funnel plots with Trim and Fill plots for LVGRS**


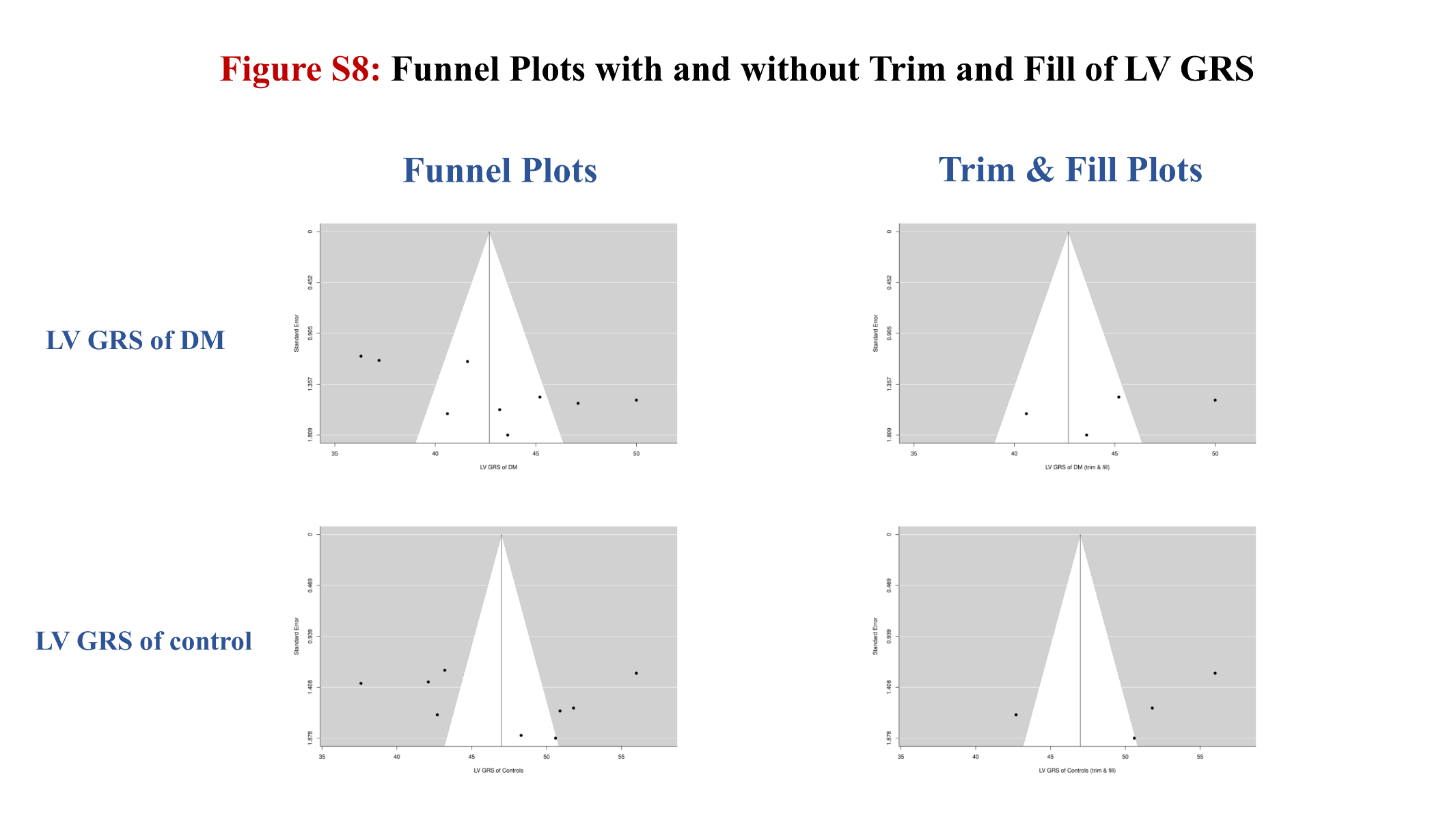


**Figure S7: Funnel plots with Trim and Fill plots for LVSR**


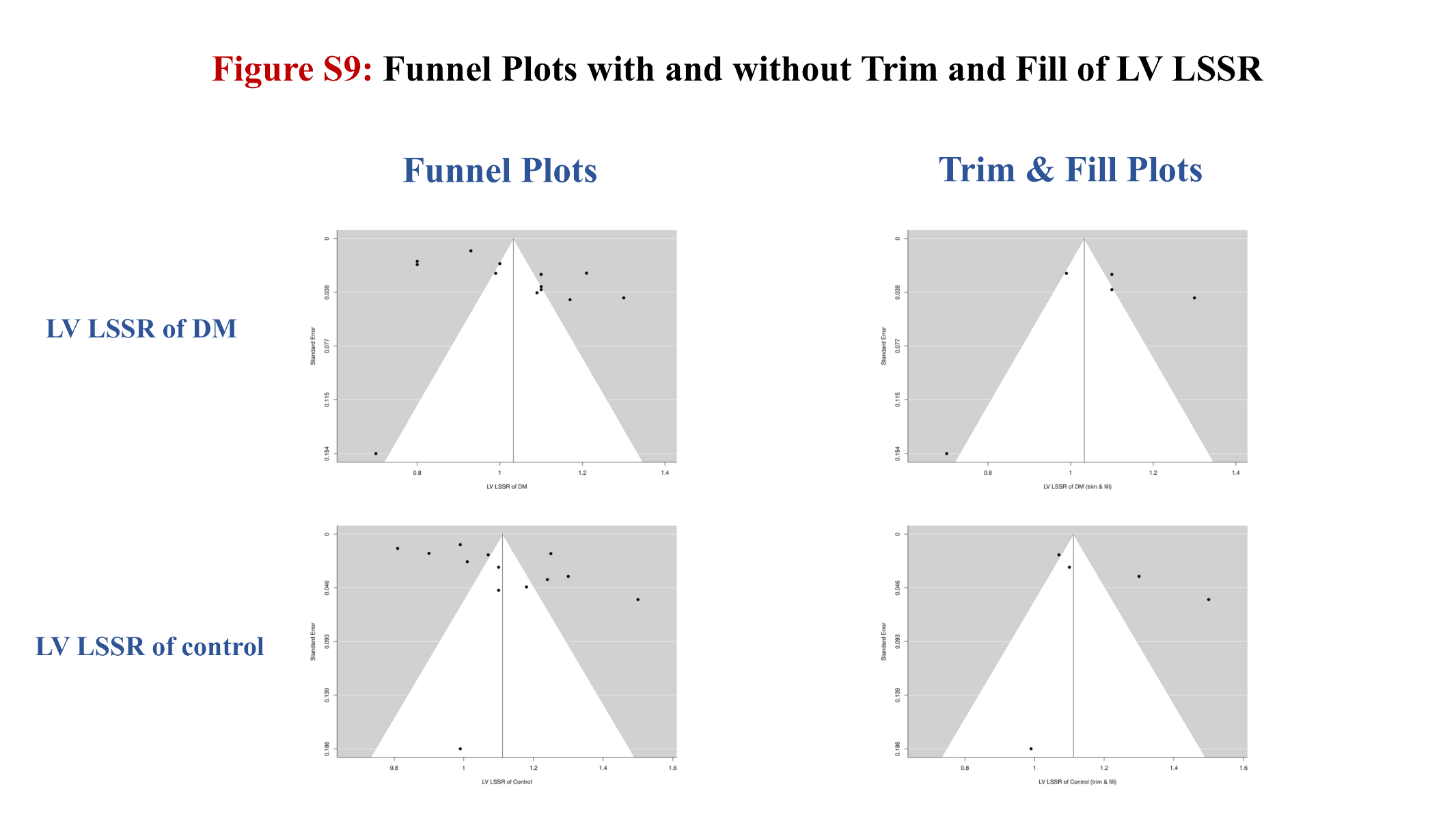


**Figure S8: Funnel plots with Trim and Fill plots for LA reservoir strain**


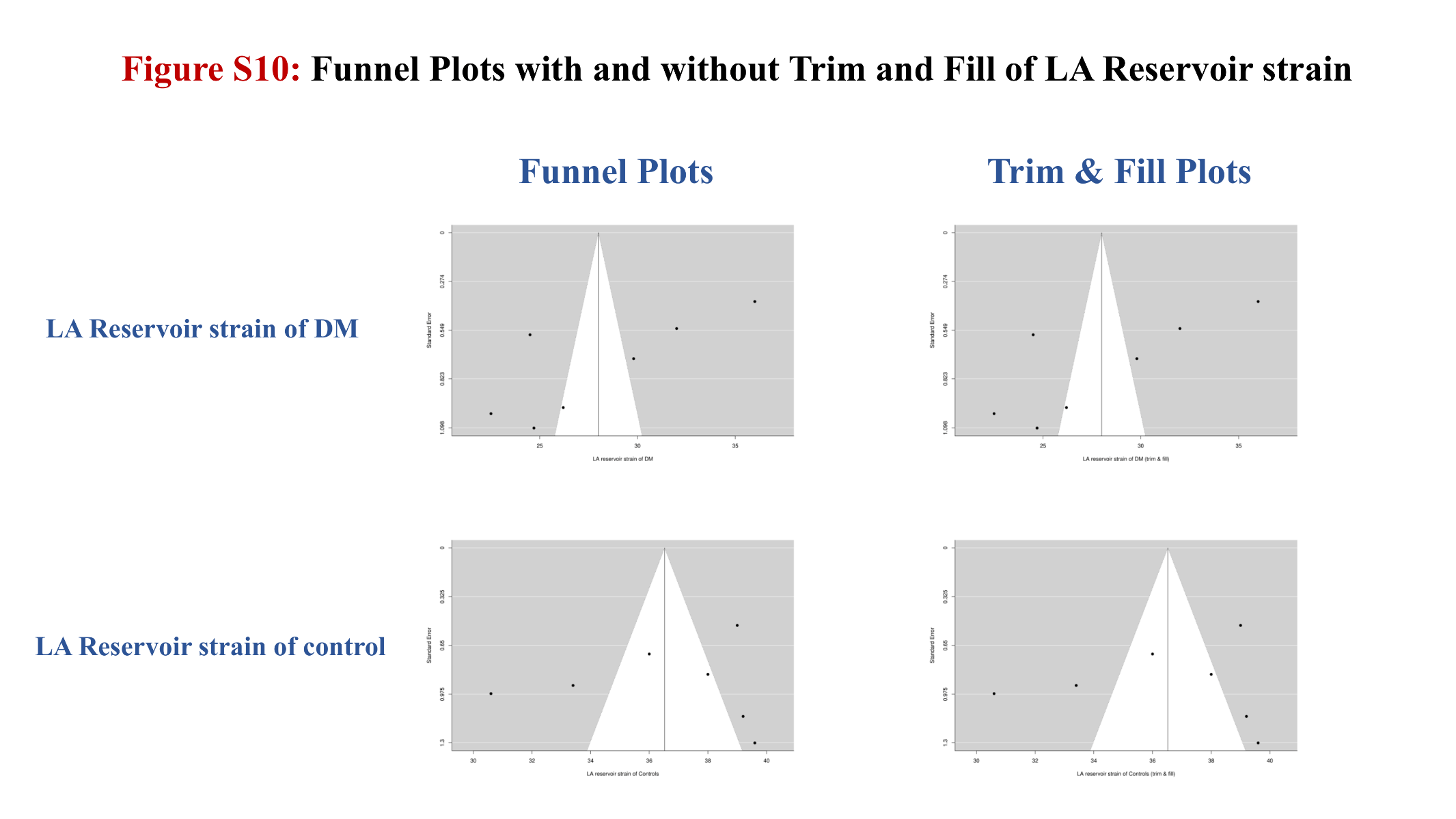


**Figure S9: Funnel plots with Trim and Fill plots for RVGLS**


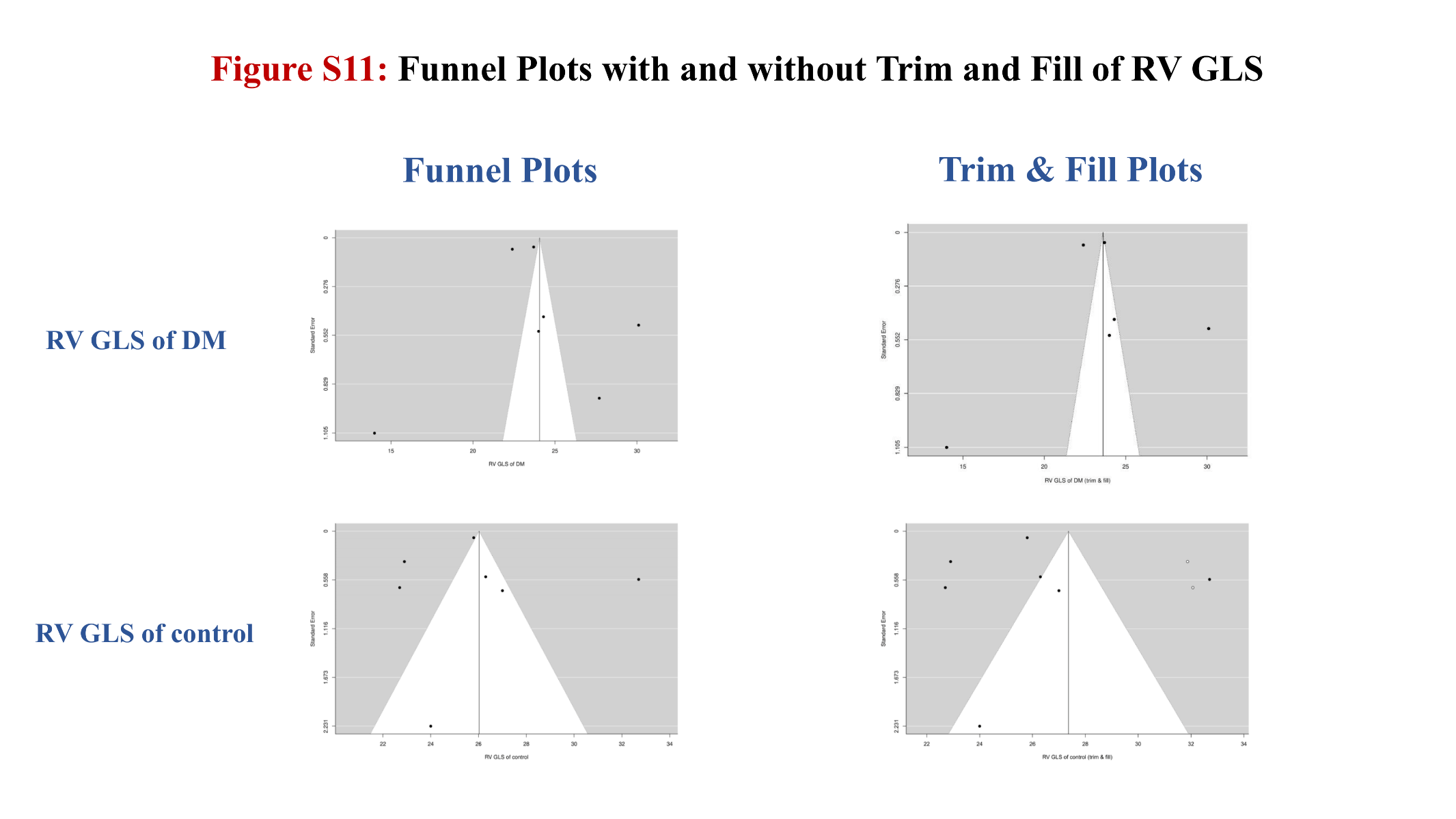


**Figure S10: Forest plots of mean LVGLS after exclusion of studies with HTN patients**


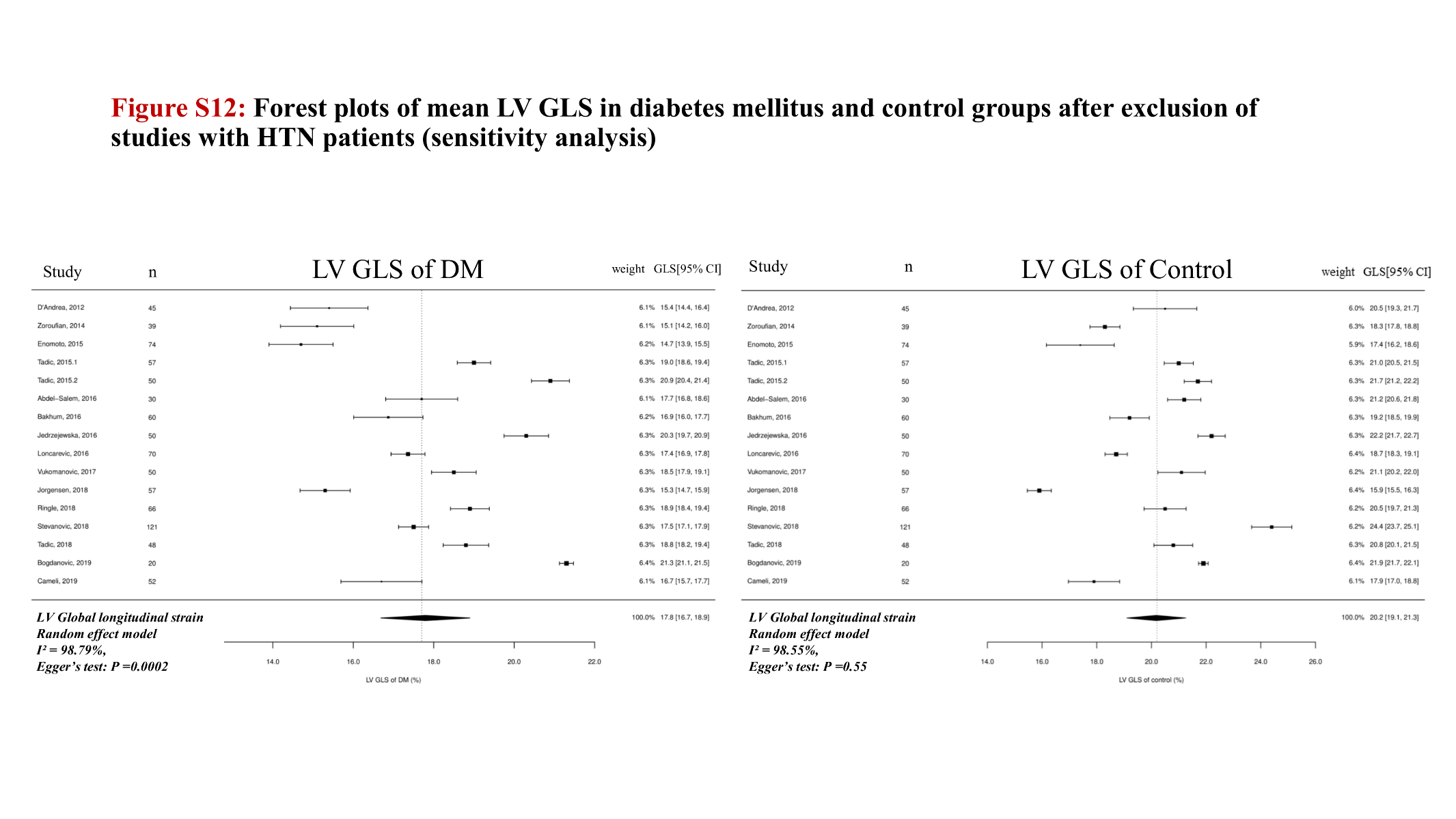


**Figure S11: Forest plot of mean LV GLS in coronary artery disease patients who have diabetes mellitus**


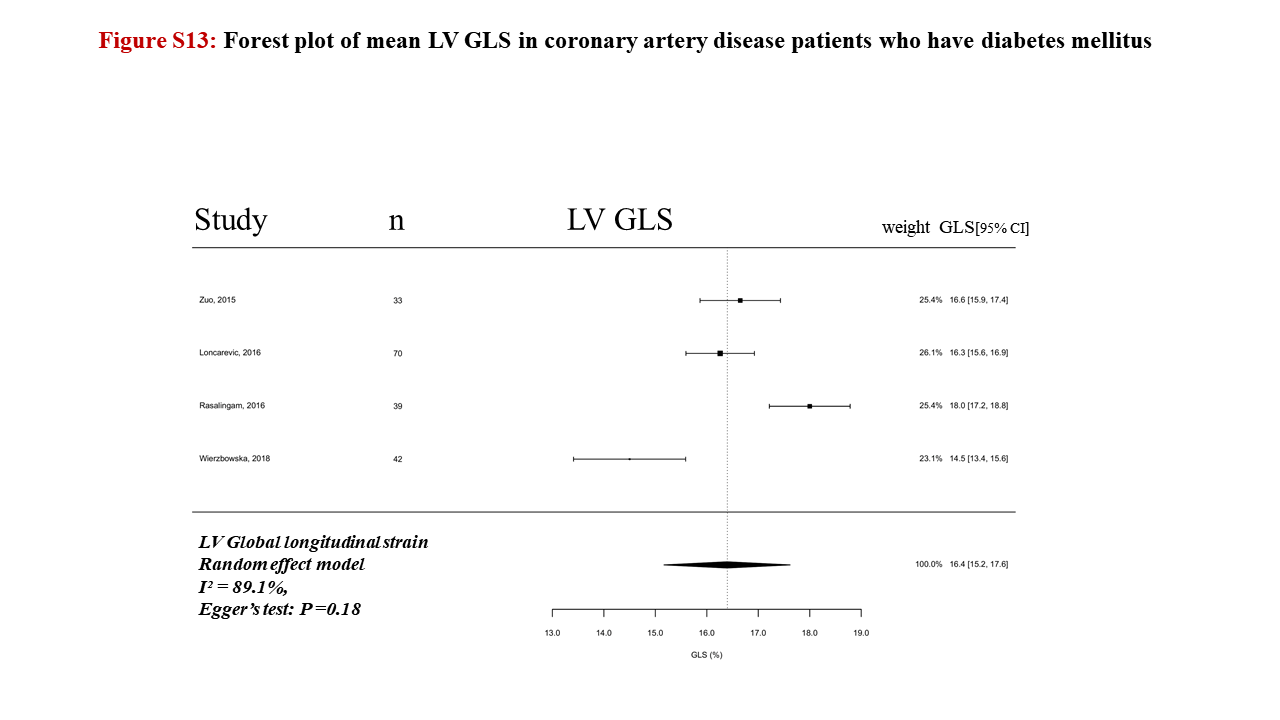

Supplement: Supplementary file 1 — Supplementary file1 (DOCX 9195 KB) [file 10554_2023_2810_MOESM1_ESM.docx]
